# Loss of *YABBY2*-Like Gene Expression May Underlie the Evolution of the Laminar Style in *Canna* and Contribute to Floral Morphological Diversity in the Zingiberales

**DOI:** 10.3389/fpls.2015.01106

**Published:** 2015-12-16

**Authors:** Kelsie Morioka, Roxana Yockteng, Ana M. R. Almeida, Chelsea D. Specht

**Affiliations:** ^1^Department of Plant and Microbial Biology, Department of Integrative Biology and the University and Jepson Herbaria, University of California at BerkeleyBerkeley, CA, USA; ^2^Corporación Colombiana de Investigación Agropecuaria (CORPOICA), Centro de Investigaciones TibaitatáTibaitatá, Colombia; ^3^Institut de Systématique, Évolution, Biodiversité, UMR 7205 Centre National de la Recherche Scientifique, Muséum National d'Histoire NaturelleParis, France; ^4^Programa de Pós-graduação em Genética e Biodiversidade, Universidade Federal da BahiaSalvador, Brazil

**Keywords:** *YABBY*, *YABBY2*, gene evolution, gene expression, floral development, plant evolution, Zingiberales, *Canna*

## Abstract

The Zingiberales is an order of tropical monocots that exhibits diverse floral morphologies. The evolution of petaloid, laminar stamens, staminodes, and styles contributes to this diversity. The laminar style is a derived trait in the family Cannaceae and plays an important role in pollination as its surface is used for secondary pollen presentation. Previous work in the Zingiberales has implicated *YABBY2*-like genes, which function in promoting laminar outgrowth, in the evolution of stamen morphology. Here, we investigate the evolution and expression of Zingiberales *YABBY2*-like genes in order to understand the evolution of the laminar style in *Canna*. Phylogenetic analyses show that multiple duplication events have occurred in this gene lineage prior to the diversification of the Zingiberales. Reverse transcription-PCR in *Canna, Costus*, and *Musa* reveals differential expression across floral organs, taxa, and gene copies, and a role for *YABBY2*-like genes in the evolution of the laminar style is proposed. Selection tests indicate that almost all sites in conserved domains are under purifying selection, consistent with their functional relevance, and a motif unique to monocot *YABBY2*-like genes is identified. These results contribute to our understanding of the molecular mechanisms underlying the evolution of floral morphologies.

## Introduction

Laminar outgrowth is a key process in the development of lateral organs, facilitating light capture and gas exchange (leaves), pollinator attraction (petals and sometimes other floral organs), and protection of the floral bud (sepals and bracts). It has been hypothesized that, at the molecular level, laminar outgrowth is promoted by the juxtaposition of abaxial and adaxial cell fates (Waites and Hudson, 1995). Numerous studies have identified and characterized the genes involved in establishing abaxial-adaxial polarity and the promotion of laminar expansion of lateral organs. In *Arabidopsis*, studies of loss-of-function and gain-of-function mutants indicate that *KANADI, ARF3/ETT*, and *ARF4* genes play roles in specifying abaxial cell fate, while *HD-ZIPIII* (*PHABULOSA, PHAVOLUTA*, and *REVOLUTA*) and *AS1/AS2* genes play roles in specifying adaxial cell fate (for more detailed review of these studies, see Bowman et al., 2002; Yamaguchi et al., 2012). *YABBY* genes are likely to act downstream in the adaxial-abaxial polarity regulatory network, promoting blade outgrowth at the abaxial-adaxial boundary (Husbands et al., 2009).

The *YABBY* gene family was believed to be specific to seed plants, but *YABBY* genes have been found in the green alga *Micromonas pusilla* (Worden et al., 2009). Since these genes have not been found in any other non-seed plants, it is unclear when this gene family evolved in plants. Members of this family encode transcription factors characterized by two domains, a zinc finger domain at the N terminus and a YABBY domain at the C terminus (Bowman and Smyth, 1999; Sawa et al., 1999a). The YABBY domain is similar in structure to the high mobility group (HMG) domain (Sawa et al., 1999a) and is necessary for DNA binding to occur (Kanaya et al., 2002). Four gene duplication events in this gene family have occurred prior to the diversification of the angiosperms (Bartholmes et al., 2012), leading to genes with both novel and redundant functions.

In angiosperms, *YABBY* genes have important roles in laminar expansion of lateral organs as well as in reproductive organ development and other processes. The *YABBY* gene family has six members in *Arabidopsis thaliana*. *FILAMENTOUS FLOWER* (*FIL*), *YABBY2*, and *YABBY3* are expressed in the abaxial domain of lateral organs and act redundantly to specify abaxial cell fate and ultimately to promote laminar outgrowth (Siegfried et al., 1999); *FIL* is also required for normal inflorescence and flower development (Chen et al., 1999; Sawa et al., 1999b). *CRABS CLAW* (*CRC*) and *INNER NO OUTER* (*INO*) have specialized functions in reproductive organ development: *CRC* is expressed in carpels and nectaries and is necessary for gynoecium elongation and nectary development (Alvarez and Smyth, 1999; Bowman and Smyth, 1999), and *INNER NO OUTER* (*INO*) is expressed in the abaxial domain of the outer integument and is required for normal outer integument development (Baker et al., 1997; Villanueva et al., 1999).

Studies in other eudicot species suggest that the abaxial expression pattern and specific function in promoting laminar outgrowth may be conserved across the eudicot lineage. In tomato, *LeYAB B* is expressed abaxially and may also specify abaxial identity (Kim et al., 2003). In *Antirrhinum majus, GRAMINIFOLIA* (*GRAM*), a *FIL*-like gene, and *PROLONGATA* (*PROL*), a *YABBY5*-like gene, are expressed abaxially and promote laminar outgrowth (Golz et al., 2004). *GRAM* has also been shown to be involved in the control of floral organ initiation and identity (Navarro et al., 2004). *TmFIL* in *Tropaelum majus* and *SrGRAM* in *Streptocarpus rexii*, both *FIL*-like genes, are also expressed abaxially (Gleissberg et al., 2005; Tononi et al., 2010).

Studies in monocots, which have focused mainly on grass species (Poales), have demonstrated varied expression patterns leading to a diversity of proposed functions. In *Zea mays* (maize), the *FIL/YAB3*-like genes *ZYB9* and *ZYB14* are expressed adaxially and may play a role in lateral outgrowth (Juarez et al., 2004). In contrast, *OsYABBY1*, a *YABBY2*-like gene from *Oryza sativa* (rice), is expressed in precursor cells that give rise to abaxial sclerenchyma in the leaves, the mestome sheath in the large vascular bundle, and sclerenchymatous cells in the palea and lemma of the flower, and is thus proposed to specify differentiation of certain cell types in rice (Toriba et al., 2007). The *FIL*-like gene *OsYABBY4* is expressed in meristems and in developing phloem and thus may be involved in vasculature development in rice (Liu et al., 2007). The gene *AaCRC* from *Asparagus asparagoides* is expressed in the abaxial region of the ovary wall and leaf phloem (Nakayama et al., 2010), while *CRC*/*DL* orthologs in Poales species are expressed throughout the carpel and in the central region of the leaf and may specify carpel identity and midrib formation (Yamaguchi et al., 2004; Ishikawa et al., 2009). It seems that divergence in expression (and possibly function) of *YABBY* genes has occurred more so in the monocot lineage than in the eudicot lineage.

Studies from the early diverging angiosperms show variation in expression as well. *AmbF1*, a *YABBY2* homolog from *Amborella trichopoda*, is expressed adaxially in all floral organs, the shoot apex, and leaves (Yamada et al., 2004). *YABBY* genes from *Cabomba caroliniana* (*CcFIL, CcYAB5, CcINO*, and *CcCRC*), however, are expressed abaxially, as in eudicots (Yamada et al., 2011). In two *Nymphaea* species, *CcINO* is expressed in the outer epidermis of the outer integument, as is *INO* in *Arabidopsis*, but is also expressed in the inner integument and the tip of the nucellus (Yamada et al., 2003). *AmbCRC* from *A. trichopoda* is expressed in the abaxial carpel, maintaining a similar expression pattern to that observed in *Arabidopsis* (Fourquin et al., 2005).

Overall, abaxial expression seems to be conserved across eudicots, and YABBY function in controlling laminar outgrowth seems to be generally conserved across angiosperms, but shifts in expression have been observed, particularly in the monocots. Based on the expression patterns of *YABBY* genes across angiosperms, the strong expression of these genes in ectopic outgrowths on both abaxial and adaxial surfaces in polarity mutants, and the later timing of *YABBY* gene expression relative to that of other genes in the polarity gene network, Husbands et al. (2009) have proposed that the ancestral function of *YABBY* genes in angiosperms may have been to promote blade outgrowth at abaxial-adaxial boundaries.

The evolution of laminarity in the androecium and gynoecium contributes to diversity in floral morphology and the evolution of plant-pollinator interactions in the tropical monocot order Zingiberales (Specht et al., 2012). This group includes the four paraphyletic banana families (Musaceae, Strelitziaceae, Lowiaceae, and Heliconiaceae) and a monophyletic group of four ginger families (Cannaceae, Marantaceae, Zingiberaceae, and Costaceae). The banana lineages are characterized by five or six fertile stamens with radial filaments, and members of Heliconiaceae have one laminar staminode, while members of the ginger clade exhibit a reduction in fertile stamen number—to one in Zingiberaceae and Costaceae and to one-half (i.e., fertile stamen with a single theca) in Cannaceae and Marantaceae—and are characterized by laminar, petaloid staminodes and fertile stamens. Members of Zingiberaceae and Costaceae possess a novel petaloid structure, the labellum, formed from two or four (Zingiberaceae) or five (Costaceae) fused laminar staminodes. The staminodes and the labellum are likely to function in pollinator attraction, making up most of the floral display in terms of showiness, coloration, and symmetry (Specht et al., 2012). In Cannaceae, the gynoecium style is also laminar and is used for secondary pollen presentation: during flower development, pollen adheres to the lateral surface of the style and is then transferred to the bill of a hummingbird pollinator once the flower opens (Glinos and Cocucci, 2011).

The evolution of laminarity in different floral organs in the Zingiberales makes this group a useful system in which to investigate the evolution of this trait and the evolution of genes involved in the abaxial-adaxial polarity gene network. Results from a recent study on the evolution of stamen morphology in the Zingiberales (Almeida et al., 2014) implicated balanced expression of abaxial-adaxial polarity genes in the formation of laminar filaments in the ginger clade, while overexpression of a *YABBY2/5* gene was implicated in the formation of radial filaments in *Musa*. Interestingly, the authors also demonstrated a similar gene expression pattern in *Brassica rapa* radial filaments, suggesting that *YABBY2/5* genes are involved in the evolution of filament morphology in angiosperms (Almeida et al., 2014).

In order to better understand the evolution of the *YABBY2* gene subfamily in the Zingiberales and its role in the evolution of laminarity in the style of Cannaceae, we isolated homologs of *Arabidopsis YABBY2* from taxa across the order and performed phylogenetic and expression analyses to investigate the evolutionary history of the gene subfamily. We identified duplication events occurring prior to the diversification of the Zingiberales and more recent, lineage-specific duplication events within the order. These data, in combination with expression data from semi-quantitative RT-PCR, were used to describe a hypothesized role of *YABBY2*-like genes in the evolution of the laminar style. We also tested for selection along branches in the *YABBY2* gene subfamily and looked for motifs characteristic of *YABBY2*-like genes.

## Materials and methods

### Plant material and cDNA synthesis

Floral buds of Zingiberales species (Table 1) were collected and immediately frozen in liquid nitrogen. Floral tissue was stored at −80°C until RNA extraction. Floral organs of young flowers from *Costus spicatus, Canna indica*, and *Musa basjoo* were also dissected in order to extract organ-specific RNA. Organ-specific material was immediately frozen in liquid nitrogen and stored at −80°C until RNA extraction. RNA extractions were performed with Plant RNA Reagent (Life Technologies) according to Yockteng et al. (2013). RNA was treated with Turbo DNase (Ambion) and cDNA was synthesized from 1.0 μg of DNase-treated RNA using iScript reverse transcriptase and oligo(dT) primers following the manufacturer's protocol (Bio-Rad Laboratories). As a control, a cDNA synthesis reaction without reverse transcriptase was set up for each sample.

**Table 1:** **Zingiberales taxa used in this study and obtained ***YABBY2***-like sequences**.

**Species**	**Location**	**NCBI accession numbers**
*Costus spicatus* (Jacq.) Sw.	2002-127 (NMNH)	KT795168-KT795183
*Canna indica* L.	2011-777 (UC Specht Lab)	KT795161
*Musa acuminata* Colla	2002-075 (NMNH)	KT795221-KT795230
*Musa basjoo* Siebold	89.0873 (UCBG)	KT795231-KT795238
*Zingiber officinale* Roscoe	MB0876 (UC Specht Lab)	KT795282-KT795284
*Strelitzia* sp. Banks	UC Specht Lab	KT795268-KT795281
*Marantochloa leucantha* (K. Schum.) Milne-Redh.	L-80.0376 (HLA)	KT795214-KT795220
*Orchidantha siamensis* K. Larsen	L-91.0308 (HLA)	KT795240-KT795247
*Phrynium oliganthum* Merr.	L-96.0226 (HLA)	KT795248-KT795251
*Schumannianthus virgatus* (Roxb.) Rolfe	L-83.0899 (HLA)	KT795258-KT795267
*Heliconia pendula* Wawra	71003-003 (McBryde)	KT795200-KT795207
*Kaempferia rubromarginata* (S. Q. Tong) R. J. Searle	L-2003.0153 (HLA)	KT795211-KT795213
*Etlingera corneri* Mood and Ibrahim	L-91.0443 (HLA)	KT795184-KT795187
*Pleuranthodium helwigii* (K. Schum.) R. M. Sm.	L-99.0492 (HLA)	KT795252-KT795257
*Globba laeta* K. Larsen	L-92.0182 (HLA)	KT795188-KT795195
*Heliconia sp*. L.	UC Specht Lab	KT795208-KT795210
*Canna jaegeriana* Urb.	MB0854 (UC)	KT795162-KT795167
*Heliconia caribaea x bihai* Lam. and L.	MB0862 (UC Specht Lab)	KT795196-KT795199
*Musa textilis* Née	1682/77 (NYBG)	KT795239

*HLA, Lyon Arboretum, Oahu, Hawaii; McBryde, McBryde Botanical Garden, Kauai, Hawaii; UCBG, University of California Botanical Garden; UC, University of California; NMNH, Smithsonian Greenhouses; NYBG, New York Botanical Garden*.

### Isolation of *YABBY2* in the zingiberales

#### Sequence retrieval

Previously published sequences from across the *YABBY* gene family were retrieved from NCBI, with representatives from early diverging angiosperms, monocots, early diverging eudicots, core eudicots, and gymnosperms. *YABBY* sequences from NCBI were blasted against the genome of *Musa acuminata* (D'Hont et al., 2012) and against the whole flower transcriptomes of *Costus spicatus, Canna indica*, and *Musa basjoo* (unpublished; Roxana Yockteng, Ana M. R. Almeida, and Chelsea D. Specht) to retrieve *YABBY* genes from these taxa. We also used RNA-seq reads from the Monocot AToL (Angiosperm Tree of Life)^1^ project to assemble transcriptomes from *Costus pulverulentus, Canna indica, Curcuma roscoeana, Orchidantha fimbriata, Heliconia collinsiana, Zingiber spectabile, Strelitzia reginae*, and *Maranta leuconeura* using Trinity r2013_08_14 (Grabherr et al., 2011). *YABBY* sequences were again blasted against these transcriptomes to retrieve *YABBY*s from these taxa. Source and accession numbers for all of the sequences used in this study are shown in Table S1.

#### Primer design

The *YABBY2* sequences from NCBI, the *Musa acuminata* genome, and the floral transcriptomes were aligned using the Geneious v5.6 algorithm (Drummond et al., 2012) and manually edited to further refine the alignment. This multiple sequence alignment was used to design forward and reverse primers at conserved sites of the gene—at the N-terminal end of the zinc finger domain and the C-terminal end of the YABBY domain, respectively—and these primers were used to amplify *YABBY2* from taxa across the Zingiberales. Zingiberales sequences then obtained through cloning were edited and added to the alignment, along with the transcriptome contigs from MonAToL, and a preliminary tree was used to design clade-specific primers of Zingiberales sequences falling in the *YABBY2* clade in order to amplify more sequences from Zingiberales taxa in each clade. All primers used in this study are found in Table S2.

#### Cloning of *YABBY2* in the Zingiberales

*YABBY2* orthologs from taxa across the eight families in the Zingiberales (Table 1) were amplified using 1.0 μl of cDNA diluted 1:50 in water, 0.3 μmol of each primer, and Phire II Hot Start DNA Polymerase (Thermo Scientific) as follows: 5 min at 98°C for initial denaturing; 40 cycles of 5 s at 98°C for denaturing, 5 s at a primer-specific annealing temperature for annealing, and 20 s at 72°C for extension; and 1 min at 72°C for final extension. PCR products were cloned into pJET1.2 blunt cloning vector (Thermo Scientific). Sequencing was performed using BigDye v3.1 on a 3730 Applied Biosystems DNA analyzer at the Museum of Vertebrate Zoology Evolutionary Genetics Laboratory at UC Berkeley.

### Phylogenetic analyses

All of the Zingiberales sequences obtained in this study and *YABBY* sequences retrieved from NCBI, the *Musa acuminata* genome, the floral transcriptomes listed above, and the MonAToL transcriptomes listed above were aligned using the Geneious v5.6 algorithm and manually edited using a codon-preserving approach to further refine the alignment. Unalignable regions outside of the zinc finger and YABBY domains were removed, resulting in a truncated alignment used for further analyses. The final truncated alignment was composed of a 132 nucleotide-long conserved region including the zinc finger domain and a 168 nucleotide-long conserved region including the YABBY domain (Figure S1), with a total alignment length of 300 nucleotides.

jModeltest 2.1.1 (Darriba et al., 2012) was used for selection of the best fit model of nucleotide evolution, and indicated the TVMef+I+G model as most appropriate for the given nucleotide alignment according to the Bayesian information criterion (BIC).

Bayesian inference was used to infer a phylogeny using MrBayes 3.2.2 (Ronquist and Huelsenbeck, 2003) in the Cipres Science Gateway^2^ using the model specified above. The MCMC was run for 10,000,000 generations and the output files were analyzed in Tracer (Rambaut et al., 2014) to check for convergence of the two chains.

A maximum likelihood phylogeny with 100 bootstrap replicates was reconstructed using PhyML 3.0 (Guindon et al., 2010) using the model specified in jModeltest.

### Selection tests

SLAC, FEL, and branch-site REL selection tests were implemented in HYPHY (Kosakovsky Pond et al., 2005; Kosakovsky Pond and Frost, 2005a) on the Datamonkey webserver (Kosakovsky Pond and Frost, 2005b) to identify sites under positive or negative (purifying) selection across the truncated multiple sequence alignment. The SLAC analysis was run using the same 300 nucleotide-long truncated multiple sequence alignment used for phylogenetic analyses, containing 133 sequences (Figure S1), and the maximum likelihood tree reconstructed using PhyML (**Figure 2**). The nucleotide model used was chosen by a Datamonkey model selection analysis. The global dN/dS value was estimated and ambiguities were averaged, and the significance level was set to 0.05. The FEL analysis was run using the same alignment, nucleotide model, and ML tree, and the significance level was set to 0.05. A branch-site REL analysis was also implemented in HYPHY using a reduced multiple sequence alignment containing 72 sequences representing all major clades in the *YABBY* gene tree, and a ML tree generated in PhyML.

We also tested for positive selection on codons along 9 branches of major clades in the *YABBY2* subfamily, in separate analyses for each branch, using branch-site model A in codeml (model = 2 and NS sites = 2) implemented in PAML (Yang, 2007). For each analysis, a likelihood ratio test (LRT) was used to determine if the difference between the likelihood scores of the alternative model (model A, in which ω for the branch of interest is estimated and the background ω is set to 0) and the null model (in which the distribution of ω is set to 1) was significantly different (degrees of freedom = 1). For each LRT, a *p*-value of 0.05 or less was required for results to be considered significant.

### Motif identification using MEME

MEME (Multiple Em for Motif Elicitation) version 4.10.0 (Bailey and Elkan, 1994) was used to identify ungapped motifs in translated YABBY2 protein sequences. Since this analysis does not require an alignment of sequences to identify conserved motifs, it could be used to search for motifs outside of the alignable regions. The “Normal” mode of motif discovery was used to search for motifs within the default width range (6-50 amino acids), and the site distribution for motifs was set to zero or one occurrence per sequence.

### Semi-quantitative RT-PCR

Reverse transcription PCR was used to determine presence or absence of expression of copies of *YABBY2* in the total flower, floral organs, and young leaves of *Costus spicatus, Musa basjoo*, and *Canna indica*. RNA extraction and cDNA synthesis were as described above. cDNA was synthesized from *C. spicatus* total flower, sepals, petals, labellum, theca, filament, gynoecium, style, and young leaves; *M. basjoo* total flower, free petal, floral tube (fused sepals and petals), theca, filament, gynoecium, style, and young leaves; and *C. indica* total flower, sepals, petals, petaloid fertile stamen, theca, staminodes, gynoecium, style, and young leaves. Primers for each gene copy were designed across intron-exon boundaries to avoid amplification of trace contaminating gDNA. All RT-PCRs were performed using 1.0 μl of cDNA diluted 1:20 in water, 0.3 μmol of copy-specific forward and reverse primers (Table S2), and Phire Hot-Start II DNA Polymerase (Thermo Scientific) as follows: 5 min at 98°C for initial denaturing followed by 35–37 cycles of 5 s at 98°C for denaturing, 5 s at a primer-specific annealing temperature for annealing, and 25 s at 72°C for elongation. The PCR products were run on 1% agarose gels.

Additionally, semi-quantitative RT-PCR was used to gauge relative levels of expression of *YABBY2* copies in the styles of *Costus spicatus, Musa basjoo*, and *Canna indica*. For each gene copy, five different reactions were run with different numbers of cycles (25, 27, 30, 32, and 35 cycles), while all other conditions were as described above. The PCR products were run on 1% agarose gels.

Products from the RT-PCRs were sequenced to confirm that the products being amplified were the targeted gene copy. At least three technical replicates were run for each reaction. *ACTIN* was used as an endogenous control; *ACTIN* primers used for each species are included in Table S2.

## Results

We obtained sequences for *YABBY2*-like genes from representative taxa across the Zingiberales in order to investigate the evolution of this gene subfamily and its potential role in the evolution of floral morphology in this order, particularly the evolution of laminarity in the gynoecium. To obtain sampling across the Zingiberales phylogeny, at least one taxon was sampled from each family. A total of 124 sequences were obtained from 19 taxa through cloning (Table 1). The sequences obtained in this study were blasted against the NCBI database to confirm that they do indeed blast to previously published *YABBY2* genes, and consensus sequences were made for sequences from the same taxa that shared at least 98% identity. Six contigs from the whole flower transcriptomes of *Costus spicatus* and *Musa basjoo* (unpublished; Roxana Yockteng, Ana M.R. Almeida, and Chelsea D. Specht) and four contigs from transcriptomes of *Canna indica* and *Costus pulverulentus* from MonAToL were also used in analyses. All sequences obtained in this study have been deposited in GenBank (KT795161-KT795284).

Almost all sequences used in phylogenetic analyses contain the zinc finger and YABBY domains characteristic of genes of the *YABBY* gene family; some sequences are missing some of the zinc finger domain and/or the YABBY domain due to primer design limitations. Sequences from at least one taxon from each family were included in the final alignment used for phylogenetic analyses. This alignment also included sequences from other monocots, core eudicots, early diverging eudicots, early diverging angiosperms, and gymnosperms.

### Evolution of *YABBY2*-like genes in the Zingiberales

The maximum likelihood reconstruction of the *YABBY* gene family is shown (**Figure 2**). Bootstrap values are shown at tree nodes, with bootstrap values greater than 50 in bold. When rooted with the gymnosperm group, the topology recovered is consistent with one of the previously proposed topologies for this gene family (Bartholmes et al., 2012), with the *YABBY2* and *YABBY5* subfamilies sister to the *INO, FIL*, and *CRC* subfamilies. The *FIL* and *CRC* subfamilies are sister to each other, and the *INO* subfamily is sister to both. The *YABBY2* and *YABBY5* subfamilies form sister clades.

Within the *YABBY2* subfamily, the *YABBY2*-like sequence from the early diverging angiosperm *Amborella trichopoda* is sister to all other *YABBY2*-like sequences from eudicots and monocots. The eudicot *YABBY2*-like sequences form a clade sister to a clade of monocot *YABBY2*-like sequences. The monocot *YABBY2*-like clade is further divided into two sister clades: one of these clades includes a clade of Poales sequences sister to three separate clades of Zingiberales sequences (*ZinYAB2-1, ZinYAB2-2*, and *ZinYAB2-3*), and the other monocot clade is composed of a clade of non-Zingiberales monocot sequences (including Poales sequences) sister to a clade of Zingiberales sequences (*ZinYAB2-4*) and one *Elaeis guineensis* sequence. Each of the Zingiberales clades includes at least one sequence from each family in the Zingiberales, with the exception of *ZinYAB2-1*, which does not have any sequences from Cannaceae. The Poales clade, the *ZinYAB2-1* clade, and the (*Elaeis guineensis* + *ZinYAB2-4*) clade are moderately well supported by the bootstrap analysis (with bootstrap values of 93, 85, and 68, respectively); bootstrap values for other clades are low, but have high or moderately high posterior probabilities in a Bayesian analysis (Figure S2).

### Expression of *ZinYAB2* genes

**Figure 1:**
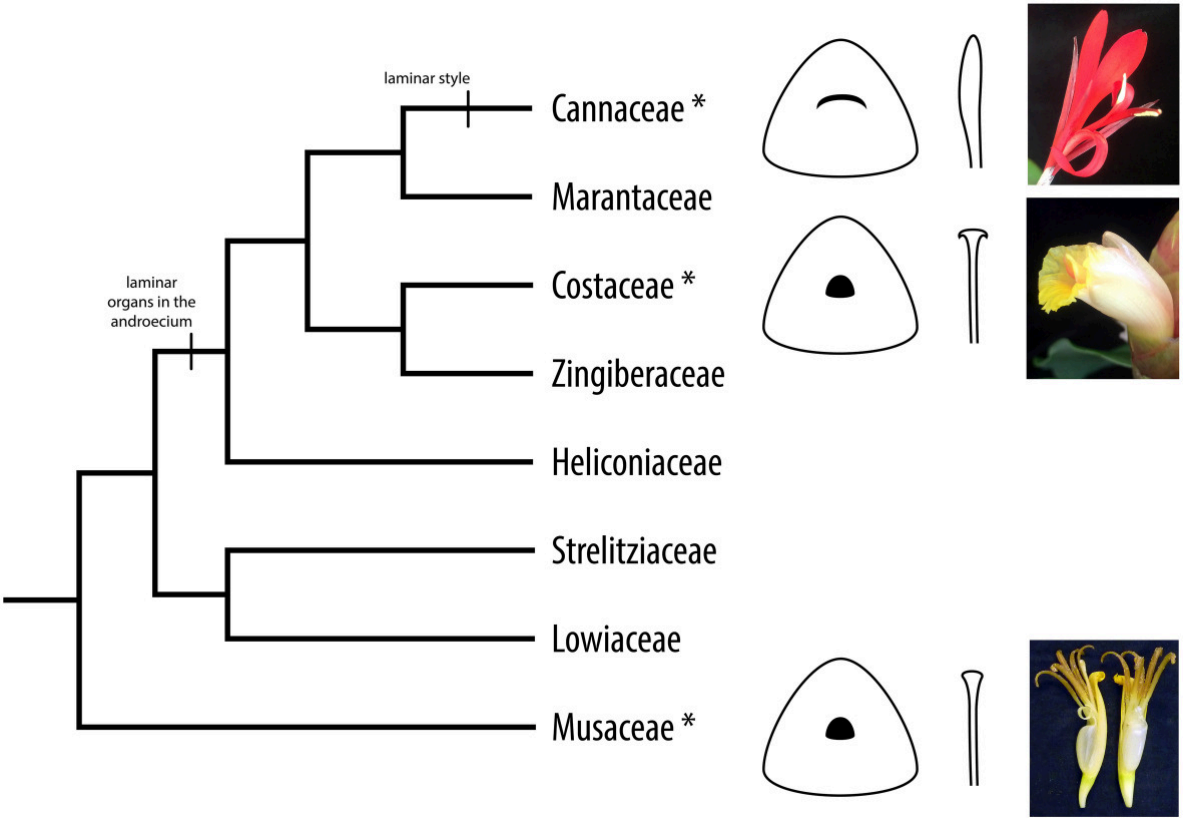
**Cladogram of the Zingiberales**. Asterisks denote taxa used for expression analyses in this study. Evolution of laminar organs is indicated: flowers in Heliconiaceae have radial filaments and one laminar staminode; flowers in the ginger clade (Cannaceae, Marantaceae, Zingiberaceae, and Marantaceae) have laminar staminodes and fertile stamens—with a staminodial labellum in Zingiberaceae and Costaceae; and flowers in Cannaceae have a laminar style. Style morphology for Cannaceae, Costaceae, and Musaceae is illustrated and flowers from representative species are shown.

Reverse transcription PCR was used to evaluate the presence and absence of expression of *YABBY2*-like genes in the floral organs and young leaves of *Canna indica* (Cannaceae), *Costus spicatus* (Costaceae), and *Musa basjoo* (Musaceae). Semi-quantitative RT-PCR was also used to gauge relative expression of *YABBY2*-like genes in the styles of these species. These taxa span the Zingiberales phylogeny and were chosen to represent the diverse floral morphology found in the order (Figure 1). Differential expression between species could indicate changes in function of *YABBY2*-like genes in the development of floral organs. Expression profiles are represented (Figure **3**) and gel images for the RT-PCRs can be found in the Supplementary data (Figure S3).

**Figure 2:**
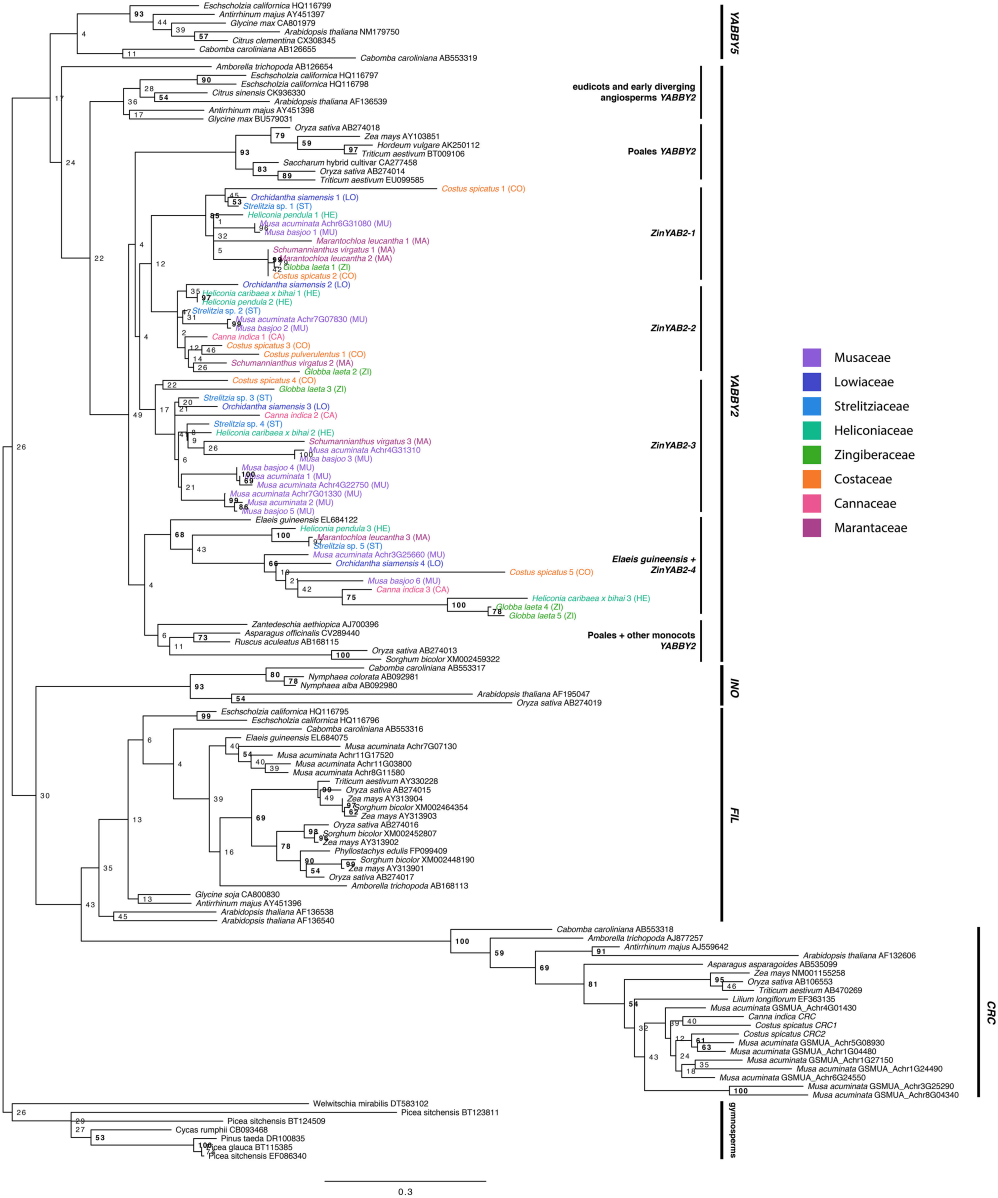
**Maximum likelihood tree of the ***YABBY***gene family rooted with gymnosperm sequences**. Bootstrap values are shown at nodes. Zingiberales *YABBY2*-like sequences are colored according to family.

**Figure 3:**
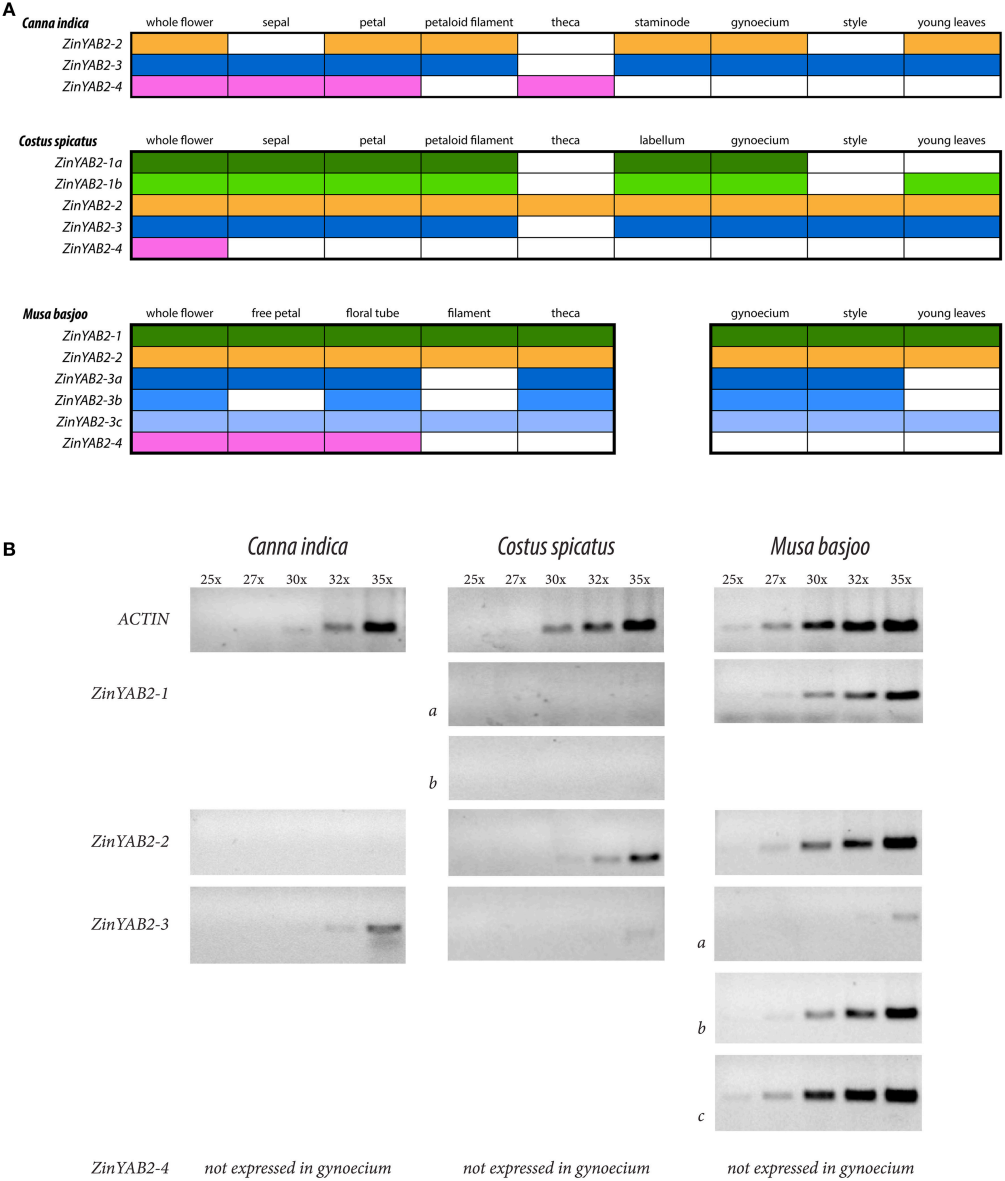
**Summary of RT-PCR results**. **(A)** Summary of RT-PCR results from *Canna indica, Costus spicatus*, and *Musa basjoo*. Each color corresponds to a different copy of *ZinYAB2*; color indicates presence and lack of color indicates absence of the gene copy. **(B)** Summary of semi-quantitative RT-PCR results from flower styles of these taxa, showing results after 25, 27, 30, 32, and 35 cycles.

In the Zingiberales, *YABBY2*-like genes show differential expression between gene copies, between species, and across floral organs and leaves (**Figure 3**). In *Musa basjoo, ZinYAB2-1* is expressed in all floral organs and in young leaves. In *Costus spicatus, ZinYAB2-1a* and *ZinYAB2-1b* are expressed in all floral organs except for stamen theca and style; *ZinYAB2-1b* is present in *C. spicatus* young leaves while *ZinYAB2-1a* is absent (**Figure 3**). In *C. indica, ZinYAB2-1* has been lost or has not been recovered in this study. *ZinYAB2-2* is expressed in all floral organs in both *M. basjoo* and *C. spicatus*, while in *C. indica* this copy is expressed in petal, petaloid filament, staminode, gynoecium, and young leaves and is absent from sepal, theca, and style (Figure **3**). In *M. basjoo*, there are three copies of *ZinYAB2-3*, resulting from duplication events in the Musaceae lineage (Figure 2): *ZinYAB2-3a* is expressed in all floral organs except filament, *ZinYAB2-3b* is expressed in all floral organs except free petal and filament, and *ZinYAB2-3c* is expressed in all floral organs (Figure 3). *ZinYAB2-3c* is the only *ZinYAB2-3* copy in *M. basjoo* that is expressed in young leaves. *ZinYAB2-3* in both *C. spicatus* and *C. indica* is expressed in young leaves and in all floral organs except theca. In *M. basjoo, ZinYAB2-4* is expressed in free petal and floral tube and is absent from filament, theca, gynoecium, style, and young leaves. In *C. spicatus, ZinYAB2-4* is present in total flower (as confirmed by three technical replicates) but was not amplified by RT-PCR for any floral organs; it is possible that this copy is so lowly expressed that it could not be amplified from floral organ tissue. In *C. indica, ZinYAB2-4* is expressed in sepal, petal, and theca, and is absent from filament, staminode, gynoecium, style, and young leaves. *ZinYAB2-4* seems to be expressed only in the flower, since there is no expression in leaves (Figure 3). Interestingly, *ZinYAB2-4* is absent from filament and gynoecium in all species considered; expression of this copy has diverged the most from that of the other *YABBY2*-like gene copies in the Zingiberales.

Across the Zingiberales, there is a pattern of reduction in the number of *YABBY2*-like gene copies expressed in the style: five gene copies are expressed in *M. basjoo* (*ZinYAB2-1, ZinYAB2-2, ZinYAB2-3a, ZinYAB2-3b*, and *ZinYAB2-3c*), two in *C. spicatus* (*ZinYAB2-2* and *ZinYAB2-3*), and one in *C. indica* (*ZinYAB2-3*). *Musa basjoo* and *C. spicatus* have the same style morphology—radial—but *YABBY2*-like expression between styles in these two species differs not only in the number of gene copies expressed, but also in the overall expression level of *YABBY2*-like genes (Figure 3). One copy, *ZinYAB2-2*, is expressed in *M. basjoo* and *C. spicatus* styles but is absent from the style of *C. indica*.

### Selection in *ZinYAB2* genes

Of the 100 sites tested, 87 sites were found to be under negative selection using the SLAC method and 90 sites were found to be under negative selection using the FEL test (*p* = 0.05). Neither test identified sites under positive selection. The branch-site REL analysis indicated that no branches are under episodic diversifying selection (*p* ≤ 0.05).

Of the nine branches of major clades in the *YABBY2* subfamily that were tested in codeml, none had codons that were found to be under positive selection once likelihood ratio tests were used to evaluate significance.

### A novel motif found in monocot *YABBY2*-like genes

**Figure 4:**
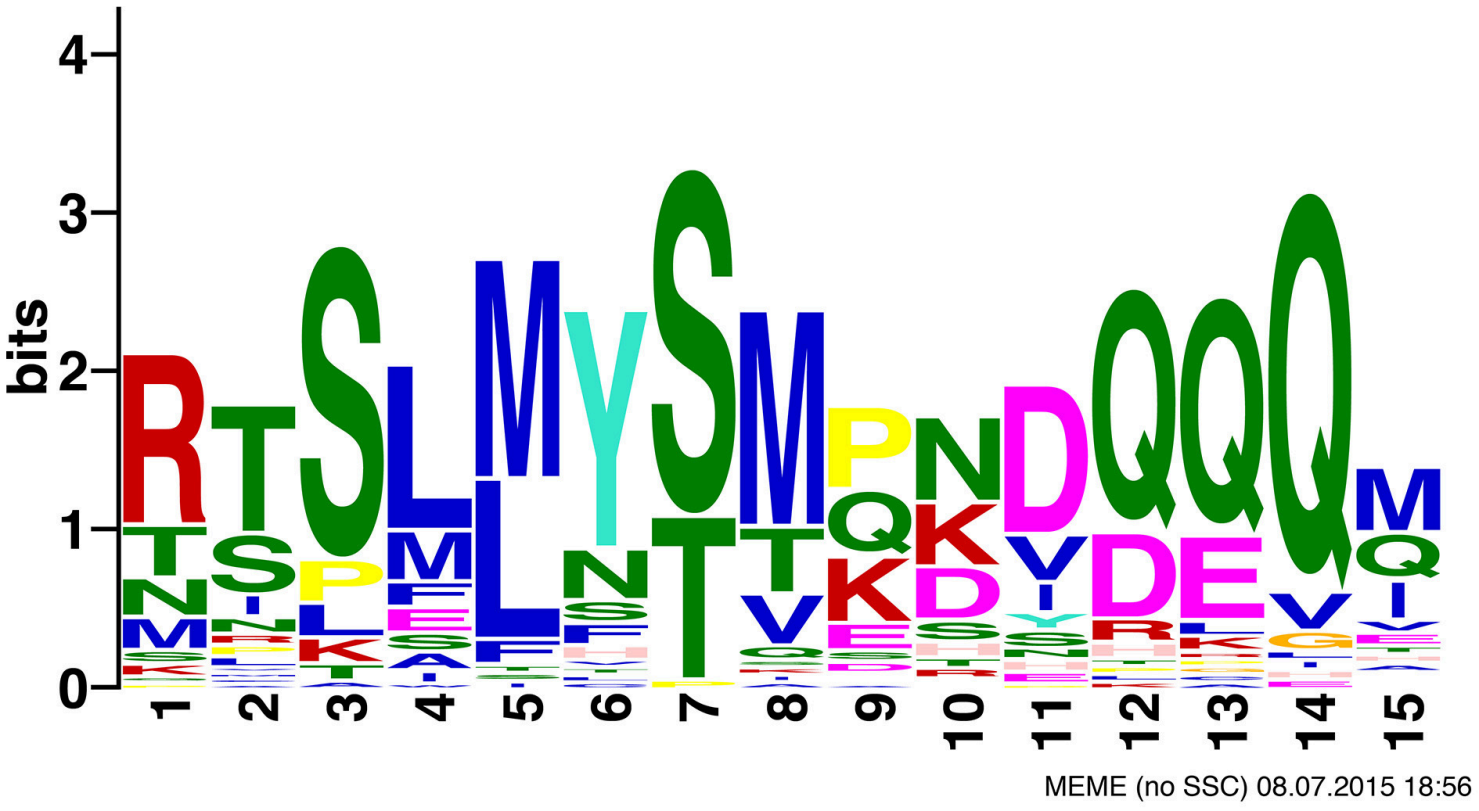
**MEME results showing a novel motif found in monocot ***YABBY2***-like sequences**.

MEME identified one motif that is conserved across most monocot *YABBY2*-like sequences. It is 15 residues in width and occurs before the YABBY domain, in-between the zinc finger and YABBY domains (Figure 4). Monocot *YABBY2*-like sequences that were identified as lacking the motif, when examined by eye, have some sequence similarity in this region, but have indels that make the conservation unrecognizable in the MEME analysis (which only recognizes ungapped motifs) or have amino acid changes that reduce similarity. This motif is not found in non-monocot *YABBY2*-like genes or in other *YABBY* subfamilies.

## Discussion

The Zingiberales is an order of tropical monocots that possesses a wide diversity of floral forms. This diversity can in part be attributed to the evolution of laminarity in the androecium and gynoecium. Members of the banana lineages (Musaceae, Heliconiaceae, Strelitziaceae, and Lowiaceae) possess radial filaments, but taxa in Heliconiaceae have single laminar staminodes. The ginger clade (Cannaceae, Marantaceae, Costaceae, and Zingiberaceae) is characterized by laminar, petaloid staminodes and stamens. Staminodes in species of Costaceae (5) and Zingiberaceae (2 or 4) are fused to form a novel laminar structure called the labellum. In Cannaceae, the gynoecium style has evolved to be laminar as well; all other families in the Zingiberales possess radial styles. Style laminarity in Cannaceae has functional importance because it facilitates secondary pollen presentation in this group: before a flower opens, pollen is transferred from the anthers to the laminar style. From there, the pollen is transferred to the bill of a hummingbird pollinator after the flower opens (Glinos and Cocucci, 2011).

In order to better understand the evolution of the laminar style in Cannaceae, we are interested in elucidating the molecular mechanisms involved in its development. In this study we have chosen to focus on one gene subfamily in particular, the *YABBY2* gene subfamily. The *YABBY* gene family encodes transcription factors involved in the abaxial-adaxial polarity molecular network responsible for laminar expansion of lateral organs (and, conversely, radialization without laminar expansion). The *YABBY2* gene subfamily was chosen because previous work has implicated the over-expression of a *YABBY2*-like gene as a mechanism for radialization in the filament of *Musa acuminata* and *Brassica rapa* (Almeida et al., 2014). To see if a similar mechanism may be involved in the evolution of the laminar style in Cannaceae, we have investigated the evolution of *YABBY2*-like genes in the Zingiberales and considered gene duplications and subsequent shifts in gene expression as a mechanism for morphological evolution.

### Evolution and diversification of *YABBY2* in the Zingiberales

The *YABBY2*-like genes from monocots form two clades, each with a non-Zingiberales monocot clade (including Poales sequences) sister to a Zingiberales clade. This suggests that there may have been a gene duplication event prior to the divergence of the Poales and Zingiberales; however, more in-depth efforts to isolate *YABBY2*-like genes from Poales species must be made to test this hypothesis. In one of these two monocot clades, the Zingiberales sequences are further divided into three separate clades, each with sequences from taxa from each the eight families in the Zingiberales—with the exception of *ZinYAB2-1*, which lacks representation from Cannaceae—suggesting that two additional gene duplication events occurred in this gene lineage before the diversification of the Zingiberales. Due to our extensive efforts to design *ZinYAB2-1*-specific primers and to clone *ZinYAB2-1* from *Canna*, we believe that *ZinYAB2-1* has likely been lost in Cannaceae, or it could not be isolated due to rapid sequence divergence. A copy of *ZinYAB2* was not found in either of the analyzed transcriptomes from *Canna*.

**Figure 5:**
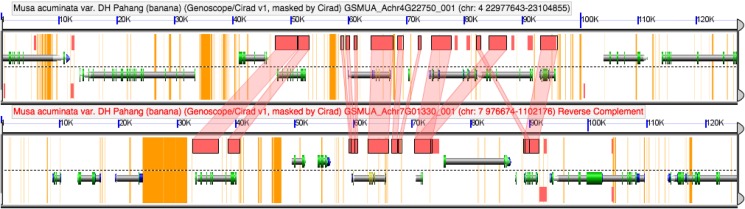
**COGE browser view of a portion of the ***Musa acuminata*** genome**. *ZinYAB2-3a* and *ZinYAB2-3c* from *Musa* are found in syntenic regions in the *Musa acuminata* genome, suggesting that they are alpha paralogs from a recent whole genome duplication.

More recent gene duplication events have occurred within the Zingiberales. Two duplication events have occurred in Musaceae in the *ZinYAB2-3* lineage, as is evidenced by *Musa acuminata* and *Musa basjoo* sequences falling sister to each other in family-specific clades. Analysis of the *Musa acuminata* genome using the Genome Evolution analysis tool (GeVo) from the CoGe platform (Lyons and Freeling, 2008) shows that two of these gene copies, *ZinYAB2-3a* and *ZinYAB2-3c*, are located in syntenic regions on chromosomes 4 and 7 and are likely alpha paralogs from a whole genome duplication event (Figure 5). The third *Musa* paralog, *ZinYAB2-3b*, may be from an older or local duplication event.

Duplication events in other Zingiberales families may have also occurred. The two *Globba laeta* (Zingiberaceae) *ZinYAB2-4* sequences are recovered as being most closely related to each other and share 87.9% identity, while the two *Costus spicatus* (Costaceae) *ZinYAB2-1* sequences are recovered as being more closely related to sequences from other species than to each other and share only 66.9% identity. This latter case is also true for the two *Marantochloa leucantha* (Marantaceae) *ZinYAB2-1* sequences and the two *Strelitzia* sp. (Strelitziaceae) *ZinYAB2-3* sequences, which share 73.5% identity and 86.9% identity, respectively. Based on sequence identity, these pairs of sequences could be allelic variants, or they could be the result of recent duplication events in these lineages.

### A novel motif found in monocot *YABBY2*-like sequences

The monocot *YABBY2*-like motif identified in the MEME analysis could be an artifact of the shared evolutionary history of these sequences, or it could be biologically relevant. Functional tests need to be performed to test whether this motif is biologically relevant and whether it results in new or altered molecular interactions for monocot *YABBY2*-like sequences. Regardless, this motif could be helpful to identify *YABBY2*-like genes from monocots in future analyses, when it is difficult to obtain support for placement of genes in the *YABBY* gene family due to the conservation in the alignable regions (the zinc finger and YABBY domains).

### Expression profiles support proposed evolutionary relationships for *ZinYAB2* gene copies

The absence of *ZinYAB2-4* from filament and gynoecium in *C. indica, C. spicatus*, and *M. basjoo* suggests that this copy is not involved in the gene network underlying laminar expansion (or radialization) in reproductive organs. The overall floral expression profiles of *ZinYAB2-1, ZinYAB2-2*, and *ZinYAB2-3* are more similar to each other than to that of *ZinYAB2-4*, which has a more restricted expression profile. These data are consistent with the hypothesis that *ZinYAB2-1, 2-2*, and *2-3* arose from two subsequent Zingiberales-specific duplications, and that the ancestor of these three copies is sister to *ZinYAB2-4* (and thus *ZinYAB2-4* is more distantly related to the other three *ZinYAB2* copies than they are to one another).

### A potential role of *YABBY2*-like genes in the evolution of the laminar style in Cannaceae

It has previously been proposed that balanced expression of genes involved in the abaxial-adaxial polarity network facilitates laminar expansion, with evidence for gene expression imbalance, through high expression of a *YABBY2*-like gene, as a mechanism for radialization in *Musa acuminata* and *Brassica rapa* filaments (Almeida et al., 2014). *Musa basjoo* and *C. spicatus* share a radial style morphology, but have different levels of expression and copy number of *YABBY2*-like genes. It is possible that there is a threshold for total *YABBY2* expression in the style at which the abaxial-adaxial polarity gene network is in balance and promotes laminar expansion, and above which the gene network is imbalanced, leading to radialization. Loss of expression of *ZinYAB2-2* (and, possibly, the loss of *ZinYAB2-1*) in the style of *C. indica* may have reduced total *YABBY2*-like gene expression to be in balance with other genes in the gene regulatory network, and thus facilitated the shift to laminar morphology in the style of *C. indica*.

Laminar expansion of the style is a derived trait in Cannaceae, and evolved separately from laminar expansion in stamens and staminodes (in Heliconiaceae and the ginger clade) and laminar expansion in sepals and petals. Perhaps the mechanism of balanced expression for laminar expansion is shared across different floral organs, but the absolute levels of expression required for laminar expansion differ in different organs; this would explain why expression differs between the laminar style and other laminar floral organs (sepal, petal, petaloid filament, and staminode) in *C. indica*.

### Future directions

To better understand the molecular mechanisms underlying the evolution of the laminar style in the Zingiberales, *in situ* hybridization experiments are needed to fully characterize the exact locations of *YABBY2*-like gene expression during development, and more gene evolution studies and expression analyses for other genes involved in the abaxial-adaxial gene regulatory network should be done. Gene knockdown (virus-induced gene silencing) or gene knockout (CRISPR) experiments are also needed to test the hypotheses proposed here about the functions of *YABBY2*-like genes in Zingiberales floral morphology. By using the evolution of style laminarity in Cannaceae as a case study, we can elucidate the mechanisms underlying the evolution of novel laminar (or radial) morphology in lateral organs. In addition, *YABBY* genes in gymnosperms have yet to be studied, and our understanding of the evolution of this gene family and thus the evolution of the abaxial-adaxial gene regulatory network would benefit greatly from gene characterization and expression studies in gymnosperms and pteridophytic vascular plants.

## Author contributions

KM, RY, AA, and CS contributed with conceptual and experimental design. KM contributed to data collection and analysis and drafted the manuscript. RY and AA contributed to data analysis and manuscript editing. CS edited the manuscript and provided financial support. All authors read and approved the final manuscript.

### Conflict of interest statement

The authors declare that the research was conducted in the absence of any commercial or financial relationships that could be construed as a potential conflict of interest.

## Footnotes

^1^http://www.botany.wisc.edu/givnish/Givnish/MonAtoL.html.

^2^http://www.phylo.org/portal2/.
