# Supplementary material for: Loss of YABBY2-Like Gene Expression May Underlie the Evolution of the Laminar Style in Canna and Contribute to Floral Morphological Diversity in the Zingiberales
Source: Front Plant Sci. 2015 Dec 16;6:1106. doi: 10.3389/fpls.2015.01106 (PMC4679924; doi:10.3389/fpls.2015.01106)
Supplement: Supplementary file 1 [file DataSheet1.DOCX]

| **TableS1.Sequences used   Species** | **in phylogenetic analyses, retrieved from NCBI &    Source** | **other sources   Accession Number** |
| --- | --- | --- |
| *Eschscholzia californica* | NCBI | HQ116799 |
| *Antirrhinum majus* | NCBI | AY451397 |
| *Glycine max* | NCBI | CA801979 |
| *Arabidopsis thaliana* | NCBI | NM179750 |
| *Citrus clementina* | NCBI | CX308345 |
| *Cabomba caroliniana* | NCBI | AB126655 |
| *Cabomba caroliniana* | NCBI | AB553319 |
| *Amborella trichopoda* | NCBI | AB126654 |
| *Eschscholzia californica* | NCBI | HQ116797 |
| *Eschscholzia californica* | NCBI | HQ116798 |
| *Citrus sinensis* | NCBI | CK936330 |
| *Arabidopsis thaliana* | NCBI | AF136539 |
| *Antirrhinum majus* | NCBI | AY451398 |
| *Glycine max* | NCBI | BU579031 |
| *Oryza sativa* | NCBI | AB274018 |
| *Zea mays* | NCBI | AY103851 |
| *Hordeum vulgare* | NCBI | AK250112 |
| *Triticum aestivum* | NCBI | BT009106 |
| *Saccharum* hybrid cultivar | NCBI | CA277458 |
| *Oryza sativa* | NCBI | AB274014 |
| *Triticum aestivum* | NCBI | EU099585 |
| *Musa acuminata* | *Musa acuminata* genome (D'Hont et al. 2012) | GSMUA_Achr6G31080 |
| *Musa acuminata* | *Musa acuminata* genome (D'Hont et al. 2012) | GSMUA_Achr7G07830 |
| *Musa acuminata* | *Musa acuminata* genome (D'Hont et al. 2012) | GSMUA_Achr4G31310 |
| *Musa acuminata* | *Musa acuminata* genome (D'Hont et al. 2012) | GSMUA_Achr4G22750 |
| *Musa acuminata* | *Musa acuminata* genome (D'Hont et al. 2012) | GSMUA_Achr7G01330 |
| *Musa acuminata* | *Musa acuminata* genome (D'Hont et al. 2012) | GSMUA_Achr3G25660 |
| *Zantedeschia aethiopica* | NCBI | AJ700396 |
| *Asparagus officinalis* | NCBI | CV289440 |
| *Ruscus aculeatus* | NCBI | AB168115 |
| *Oryza sativa* | NCBI | AB274013 |
| *Sorghum bicolor* | NCBI | XM002459322 |
| *Cabomba caroliniana* | NCBI | AB553317 |
| *Nymphaea colorata* | NCBI | AB092981 |
| *Nymphaea alba* | NCBI | AB092980 |
| *Arabidopsis thaliana* | NCBI | AF195047 |
| *Oryza sativa* | NCBI | AB274019 |
| *Eschscholzia californica* | NCBI | HQ116795 |
| *Eschscholzia californica* | NCBI | HQ116796 |
| *Cabomba caroliniana* | NCBI | AB553316 |
| *Elaeis guineensis* | NCBI | EL684075 |
| *Musa acuminata* | *Musa acuminata* genome (D'Hont et al. 2012) | GSMUA_Achr7G07130 |
| *Musa acuminata* | *Musa acuminata* genome (D'Hont et al. 2012) | GSMUA_Achr11G17520 |
| *Musa acuminata* | *Musa acuminata* genome (D'Hont et al. 2012) | GSMUA_Achr11G03800 |
| *Musa acuminata* | *Musa acuminata* genome (D'Hont et al. 2012) | GSMUA_Achr8G11580 |
| *Triticum aestivum* | NCBI | AY330228 |
| *Oryza sativa* | NCBI | AB274015 |
| *Zea mays* | NCBI | AY313904 |
| *Sorghum bicolor* | NCBI | XM002464354 |
| *Zea mays* | NCBI | AY313903 |
| *Oryza sativa* | NCBI | AB274016 |
| *Sorghum bicolor* | NCBI | XM002452807 |
| *Zea mays* | NCBI | AY313902 |
| *Phyllostachys edulis* | NCBI | FP099409 |
| *Sorghum bicolor* | NCBI | XM002448190 |
| *Zea mays* | NCBI | AY313901 |
| *Oryza sativa* | NCBI | AB274017 |
| *Amborella trichopoda* | NCBI | AB168113 |
| *Glycine soja* | NCBI | CA800830 |
| *Antirrhinum majus* | NCBI | AY451396 |
| *Arabidopsis thaliana* | NCBI | AF136538 |
| *Arabidopsis thaliana* | NCBI | AF136540 |
| *Cabomba caroliniana* | NCBI | AB553318 |
| *Amborella trichopoda* | NCBI | AJ877257 |
| *Antirrhinum majus* | NCBI | AJ559642 |
| *Arabidopsis thaliana* | NCBI | AF132606 |
| *Asparagus asparagoides* | NCBI | AB535099 |
| *Zea mays* | NCBI | NM001155258 |
| *Oryza sativa* | NCBI | AB106553 |
| *Triticum aestivum* | NCBI | AB470269 |
| *Lilium longiflorum* | NCBI | EF363135 |
| *Musa acuminata* | *Musa acuminata* genome (D'Hont et al. 2012) | GSMUA_Achr4G01430 |
| *Canna indica* | Monocot AToL project (www.botany.wisc.edu/givnish/monocotatol.htm) | |
| *Costus spicatus* | unpublished transcriptome; Roxana Yockteng, Ana Almeida, and Chelsea Specht | |
| *Costus spicatus* | unpublished transcriptome; Roxana Yockteng, Ana Almeida, and Chelsea Specht | |
| *Musa acuminata* | *Musa acuminata* genome (D'Hont et al. 2012) | GSMUA_Achr5G08930 |
| *Musa acuminata* | *Musa acuminata* genome (D'Hont et al. 2012) | GSMUA_Achr1G04480 |
| *Musa acuminata* | *Musa acuminata* genome (D'Hont et al. 2012) | GSMUA_Achr1G27150 |
| *Musa acuminata* | *Musa acuminata* genome (D'Hont et al. 2012) | GSMUA_Achr1G24490 |
| *Musa acuminata* | *Musa acuminata* genome (D'Hont et al. 2012) | GSMUA_Achr6G24550 |
| *Musa acuminata* | *Musa acuminata* genome (D'Hont et al. 2012) | GSMUA_Achr3G25290 |
| *Musa acuminata* | *Musa acuminata* genome (D'Hont et al. 2012) | GSMUA_Achr8G04340 |
| *Welwitschia mirabilis* | NCBI | DT583102 |
| *Picea sitchensis* | NCBI | BT123811 |
| *Picea sitchensis* | NCBI | BT124509 |
| *Cycas rumphii* | NCBI | CB093468 |
| *Pinus taeda* | NCBI | DR100835 |
| *Picea glauca* | NCBI | BT115385 |
| *Picea sitchensis* | NCBI | EF086340 |
|  |  |  |

**Table S2. Primers used in this study***These primers are from Almeida et al. 2014.

| **Primer name** | **Taxa** | **Sequence (5’🡪 3’)** |
| --- | --- | --- |
| Zin-YABBY2-For | Zingiberales | TGC AAC TTC TGC AAC ACC AT |
| Zin-YABBY2-Rev | Zingiberales | AAA GTG CGC CCA ATT TTT AG |
| Musa-acuminata-YABBY2-For | *Musa acuminata* | ATG TCK GCC CAR RTT CCW C |
| Musa-acuminata-YABBY2-Rev | *Musa acuminata* | AAG AAR CTC TGA GCT TTY TG |
| Zin-YABBY2-A-For | Zingiberales | CAC GGT TAA CAT TCC ATG C |
| Zin-YABBY2-B-For | Zingiberales | TGT GGR CTY TGT GCT AAT WTA CTG |
| Zin-YABBY2-C-For | Zingiberales | CCT GGC RAC AGC TTG TTC |
| Zin-YABBY2-D-For | Zingiberales | ACC ATG CTT GCG RTT WAT GT |
| Zin-YABBY2-E-Rev | Zingiberales | GTG CTR AAT GCT TST YTG TG |
| Zin-YABBY2-F-For | Zingiberales | AGA CTG TTG AAC TTG GTG ACG |
| Zin-YABBY2-G-Rev | Zingiberales | AAT ATC AGG RTT GTT RGC TTT TAT CC |
| Strelitzia-YABBY2-Rev | *Strelitzia sp.* | GAA TGC TTC CTT RTG GCT AAT G |
| Musa-basjoo-YABBY2-Rev | *Musa basjoo* | TTT TTR GCT GCW GTG CYG A |
| Costus-spicatus-ZinYAB2-2-For | *Costus spicatus* | TGC AAC TTC TGC AAC ACC AY |
| Costus-spicatus-ZinYAB2-2-Rev | *Costus spicatus* | CCT TYG TAT CTC CTC CCT GAT AAA C |
| Musa-basjoo-ZinYAB2-4-For | *Musa basjoo* | GTG GRC TYT GTG CTA ATW TAC TG |
| Musa-basjoo- ZinYAB2-4-Rev | *Musa basjoo* | AAG TGY GCC CAR TTT TTR GC |
| RTPCR-Canna-indica-ZinYAB2-4-For | *Canna indica* | CCA CTC GAT CAG AAT GAG AAG G |
| RTPCR-Canna-indica-ZinYAB2-4-Rev | *Canna indica* | CAG TTG CTG CAG GTT GGA T |
| RTPCR-Canna-indica-ZinYAB2-2-For | *Canna indica* | GAT CTT CAG AAC AAC TTT GGA TTT C |
| RTPCR-Canna-indica-ZinYAB2-2-Rev | *Canna indica* | TCG TAT CTC CTC CCT GAT AAA CC |
| RTPCR-Canna-indica-ZinYAB2-3-For | *Canna indica* | TTC CAG GCC TGC AAC ATA G |
| RTPCR-Canna-indica-ZinYAB2-3-Rev | *Canna indica* | CTT TGT ATT TCC TCC TTG ATA AAC TTG |
| RTPCR-Costus-spicatus-ZinYAB2-4-For | *Costus spicatus* | TCA GAG CTT TCA GAA TCA GAA CC |
| RTPCR-Costus-spicatus-ZinYAB2-4-Rev | *Costus spicatus* | CTT AAG CCT CTG CAT CTC TTC CT |
| RTPCR-Costus-spicatus-ZinYAB2-1b-For | *Costus spicatus* | CTA CCA GGT CTA TAG CCT TGA ATC TC |
| RTPCR-Costus-spicatus-ZinYAB2-1b-Rev | *Costus spicatus* | ATC CTT TGA ATT TCT TCC TTG ATA AAC |
| RTPCR-Costus-spicatus-ZinYAB2-1a-For | *Costus spicatus* | AAA ATT TCC AGG CAC AAG TGA G |
| RTPCR-Costus-spicatus-ZinYAB2-1a-Rev | *Costus spicatus* | TTT GAA TCT CCT CTT TGA TGA ACT T |
| RTPCR-Costus-spicatus-ZinYAB2-3-For | *Costus spicatus* | AGG CCC GGA ACG TAG AAT |
| RTPCR-Costus-spicatus-ZinYAB2-3-Rev | *Costus spicatus* | TCC TTT GTA TTT CCT CCT TTA TGA AC |
| RTPCR-Costus-spicatus-ZinYAB2-2-For | *Costus spicatus* | TCT ACA GTT GCA GAA CCA GTA CAC C |
| RTPCR-Costus-spicatus-ZinYAB2-2-Rev | *Costus spicatus* | AAC ACG TTG CCT CTT CTC TGG |
| RTPCR-Musa-basjoo-ZinYAB2-4-For | *Musa basjoo* | TCC GTG AAT CTG CTG GAA G |
| RTPCR-Musa-basjoo-ZinYAB2-4-Rev | *Musa basjoo* | CTC TGT TGG TTG GAT GAG CTG |
| RTPCR-Musa-basjoo-ZinYAB2-3a-For | *Musa basjoo* | TCC TTT CCA AGA TTT CCA GAC C |
| RTPCR-Musa-basjoo-ZinYAB2-3a-Rev | *Musa basjoo* | TGA TAT CTG GAT TCT TAG CCT TTA TC |
| RTPCR-Musa-basjoo-ZinYAB2-1-For | *Musa basjoo* | ATT GCA AGA TCT TCA GAC CTA CG |
| RTPCR-Musa-basjoo-ZinYAB2-1-Rev | *Musa basjoo* | CTA GGA TTA TTT GCC TTG ATC CTT T |
| RTPCR-Musa-basjoo-ZinYAB2-3b-For | *Musa basjoo* | ACC CTT CTC AAG GCT TTC AGA TAT |
| RTPCR-Musa-basjoo-ZinYAB2-3b-Rev | *Musa basjoo* | CTT TTA TCC TTT GTA TTT CCT CCT TG |
| RTPCR-Musa-basjoo-ZinYAB2-2-For | *Musa basjoo* | TCT TCA AGA TTT CCA GGT GCA T |
| RTPCR-Musa-basjoo-ZinYAB2-2-Rev | *Musa basjoo* | TTT GTA TCT CCT CCC TGA TAA ACC |
| RTPCR-Musa-basjoo-ZinYAB2-3c-For (*MaYABBY2/5* F)* | *Musa acuminata* | AGC ATT GTA GCA GTG CGA TG |
| RTPCR-Musa-basjoo-ZinYAB2-3c-Rev (*MaYABBY2/5* R)* | *Musa acuminata* | GGA CGC ATA GGC AGC ATA AT |
| RTPCR-Costus-spicatus-ACTIN-For (*CsACTIN1* F)* | *Costus spicatus* | GCA TGA GCA AGG AGA TCA CA |
| RTPCR-Costus-spicatus-ACTIN-Rev (*CsACTIN1* R)* | *Costus spicatus* | CAA ACA TGA CTT GGG TGT GC |
| RTPCR-Musa-basjoo-ACTIN-For | *Musa basjoo* | AGG TAT CGT GTT GGA TTC TGG AG |
| RTPCR-Musa-basjoo-ACTIN-Rev | *Musa basjoo* | TCT CCT TGA TGT CAC GTA CAA TTT C |
| RTPCR-Canna-indica-ACTIN-For | *Canna indica* | GAT ATT CCT TCA CAA CCA GTG CT |
| RTPCR-Canna-indica-ACTIN-Rev | *Canna indica* | GCA CCT CTG GGC ATC TAA AG |

**Figure S1.** Nucleotide alignment used for phylogenetic analyses.

**Figure S2.** Bayesian 50% majority rule consensus tree of the *YABBY* gene family rooted with gymnosperm sequences. Posterior probabilities are shown at nodes. Zingiberales *YABBY2*-like sequences are colored according to family.

**Figure S3.** Gel images showing RT-PCR results across floral organs in *Canna indica*, *Costus spicatus*, and *Musa basjoo*. WF: whole flower, se: sepals, pe: petals, pfil: petaloid filament, th: theca, sto: staminode, gyn: gynoecium, veg: young leaves (vegetative), lab: labellum, fil: filament, fp: free petal, ft: floral tube (fused sepals and petals).
