# Supplementary material for: Loss of YABBY2-Like Gene Expression May Underlie the Evolution of the Laminar Style in Canna and Contribute to Floral Morphological Diversity in the Zingiberales
Source: Front Plant Sci. 2015 Dec 16;6:1106. doi: 10.3389/fpls.2015.01106 (PMC4679924; doi:10.3389/fpls.2015.01106)
Supplement: Supplementary file 2 [file Image1.pdf]

133 300
Amborella_trichopoda_AB126654
GAGCACGTCTGCTATGTTCAGTGCAACCTATGCAACACCATTTTAGCGGTTAGTGTTCCAGGAAGCTGCTTG---
---TTCGGTATTGTAACAGTAAGATGCGGGCATTGCACCAATCTGCTCTCTATGAATAATCGACCACCAGAG---
AAGAGGCAACGCGTTCCATCAGCTTACAACCGCTTTATAAAAGAGGAAATCCAAAGGATCAAGGCTAGGAATCCC
GAGATAACCCATAGGGAGGCCTTCAGCACTGCAGCAAAGAATTGGGCTCACTTCCCTCATCTTCATTATGGATTG
Amborella_trichopoda_AB168113
GAGCAGTTGTGCTATGTCCACTGCAACTTTTGCGACACTGTCCTCGCAGTGAGCGTTCCATGCAGCAGCCTA---
---TTCAAGATGGTTACAGTGAGATGCGGTCATTGCACCAATGTTCTCTCCGTCGATAGCCGTCCTCCGGAG---
AAGAGACAAAGAGTTCCCTCGGCGTACAACCGCTTTATCAAGGAAGAGATCCAACGAATCAAAGCGGGAAACCCG
GACATCACCCACAGGGAAGCGTTCAGCACTGCGGCCAAGAACTGGGCTCACTTTCCACACATCCATTTCGGTCTT
Amborella_trichopoda_AJ877257
GACCATCTTTGCTACGTTCGCTGCAACTTCTGCGACACCCTTCTCGCTGTTGGTGTTCCATGCAGAAGGTTA---
---ATGGACACAGTGACAGTGAAGTGTGGGCATTGCAGCCAT---CTCTCATTCCTCGTAAAGCCTCCTGAG---
AAGAAACACAGGCTCCCTTCAGCTTACAATCGGTTCATGAAGGAGGAGATAAAGAGAATCAAAGCTGGAAACCCG
GAAATACCACATAGAGAAGCGTTCAGCATGGCTGCAAAGAATTGGGCCAGGTTCGATCCTCAACTGCTGCATGGC
Antirrhinum_majus_AY451398
GAATGTGTTTGTTACGTTCACTGCAACTTCTGCAACACCATTCTAGCGGTGAGTGTTCCTTGCAGCAATATG---
---TTTACGATTGTGACTGTGAGATGTGGGCATTGTGCAAATCTGCTGTCTGTTAATATTCGTCCTCCAGAG---
AAAAGACAACGTGTCCCATCAGCATATAACCGATTCATAAAGGAGGAGATTCAGAGGATAAAGGCTGGCAATCCA
GAAATTAGTCACAGGGAAGCTTTTAGCACAGCTGCAAAAAATTGGGCACATTTCCCTCATATTCATTTTGGACTA
Antirrhinum_majus_AJ559642
GAGCATCTTTGCTATGTCCGTTGCAACTTCTGCAGCACTGTTCTTGCGGTTGGGATTCCATGCAAGAGGCTG---
---ATGGACACAGTGACTGTGAAATGTGGGCACTGCAGCAAT---CTCTCATTTCTCGTGAAACCTCCTGAG---
AAGAAGCACAGGCTTCCATCAGCCTACAATCGGTTCATGAAAGAGGAGATACAGCGTATCAAAGCAGCCAATCCG
GAGATACCACATCGAGAGGCTTTCAGTGCAGCTGCAAAAAATTGGGCTAGGTACATTCCAAACACTCCACCACCA
Antirrhinum_majus_AY451396
GAGCAGCTCTGTTACGTCCATTGCAACTTTTGTGACACTGTCCTCGCGGTGAGTGTTCCTTGCACTAGCTTG---
---ATCAAGACTGTGACTGTGAGATGTGGGCATTGCACCAACCTTCTGTCCGTGAACAACAGACCCCCGGAG---
AAAAGACAGAGAGTGCCATCAGCTTACAACCGCTTCATAAAGGACGAGATCCAACGTATCAAAGCTGGAAACCCT
GATATAAGTCACAGGGAGGCCTTCAGTGCCGCAGCTAAAAATTGGGCCCACTTCCCACACATTCACTTTGGCCTC
Antirrhinum_majus_AY451397
GAGCAACTTTGCTACATTTCTTGCAACTTCTGCAGTATTGTTCTTGCGGTAAGTGTTCCATGCAGTAGCTTG---
---TTTGATGTTGTGACAGTTCGATGTGGGCACTGTACCAATCTATGGAGTGTGAATAATCGACCCCCTGAG---
AAGAGGCAACGAGTGCCATCTGCATATAACCAGTTCATAAAAGAAGAGATTCAAAGAATTAAGGCCAATAATCCA
GAAATAAGCCACAGAGAAGCATTCAGCACTGCTGCCAAAAATTGGGCACACTTTCCTCACATTCATTTTGGGCTG
Arabidopsis_thaliana_AF132606
GAACATCTCTACTACGTCCGGTGTAGCATCTGCAACACCATCCTCGCGGTTGGGATACCATTGAAGAGAATG---
---CTTGACACGGTAACGGTGAAATGCGGCCATTGTGGTAAC---CTCTCGTTTCTCGTCAAACCTCCTGAG---
AAGAAGCAGAGGCTCCCATCTGCATACAACCGCTTCATGAGGGATGAGATCCAACGCATCAAAAGTGCCAATCCG
GAAATACCACACCGTGAAGCTTTCAGTGCTGCTGCCAAAAATTGGGCTAAGTACATACCCAACTCTCCTACTTCC
Arabidopsis_thaliana_AF136538
GACCATCTCTGCTATGTCCAATGCAACTTTTGCCAAACCATCCTTGCGGTTAATGTTCCTTACACAAGCTTG---
---TTCAAGACCGTAACTGTCCGATGTGGTTGCTGTACCAATCTCCTTTCGGTGAACAACCGCCCTCCAGAG---
AAAAGACAGAGAGTCCCATCCGCATATAACCGATTCATCAAGGAGGAGATCCAACGTATCAAAGCTGGTAATCCT
GATATAAGCCACAGAGAAGCCTTTAGTGCTGCTGCCAAGAATTGGGCCCACTTCCCCCACATACACTTCGGGCTC

Arabidopsis_thaliana_AF195047
GGGCAGATTTGCCATGTCCAGTGTGGTTTTTGCACCACTATTTTGCTGGTGAGTGTACCGTTTACAAGCTTG---
---TCAATGGTGGTGACTGTGAGATGTGGGCATTGCACAAGCCTTCTCTCTGTCAATAATAAACCACCTGAG---
AAGCGACAAAGAGCTCCTTCAGCTTACAATTGCTTCATCAAGGAAGAGATCAGGAGGTTAAAGGCTCAGAATCCA
AGCATGGCTCACAAGGAAGCTTTCAGCTTAGCTGCCAAAAATTGGGCCCATTTTCCTCCAGCTCACAACAAGAGA
Arabidopsis_thaliana_AF136539
GAGCGTGTTTGCTATGTCCACTGCAGCTTCTGCACCACGATTTTAGCGGTAAGTGTACCATACGCAAGTTTG---
---TTCACACTTGTGACGGTGAGATGTGGCCATTGTACCAATTTGCTATCCCTCAACATTCGCCCACCGGAG---
AAAAGACAACGTGTTCCTTCGGCCTACAACAGATTCATCAAGGAGGAAATCCAAAGGATTAAGGCTTGCAATCCA
GAGATTAGCCACCGTGAGGCATTTAGCACTGCTGCTAAAAATTGGGCACATTTTCCTCACATTCACTTTGGATTA
Arabidopsis_thaliana_AF136540
GACCAGCTCTGTTACGTCCATTGCAGCTTCTGCGACACTGTCCTTGCTGTGAGTGTTCCTCCGAGTAGTTTG---
---TTCAAGACGGTGACGGTCAGATGCGGCCACTGTTCGAACCTTTTGTCGGTGACCAATAGACCCCCAGAG---
AAGCGACAAAGAGTACCATCTGCATACAACCGATTCATCAAAGAGGAGATCCAACGTATAAAGGCAGGCAACCCT
GATATCAGCCACAGAGAAGCCTTCAGTGCTGCTGCCAAAAACTGGGCTCATTTCCCTCACATACACTTTGGACTC
Arabidopsis_thaliana_NM179750
GAACAGCTCTGCTACATCCCTTGCAACTTTTGCAACATAATTCTTGCGGTGAATGTCCCATGCAGCAGCCTG---
---TTCGACATCGTGACCGTCCGATGCGGTCACTGCACCAATCTGTGGTCTGTAAACAACCGTCCTCCGGAG---
AAGCGGCAGCGAGTACCTTCTGCGTACAATCAATTCATAAAAGAGGAAATTCAGAGGATTAAGGCGAATAATCCA
GACATAAGCCACAGAGAAGCATTCAGCACTGCTGCCAAGAATTGGGCACACTTTCCTCATATTCACTTTGGTCTA
Asparagus_asparagoides_AB535099
GAACACCTGTGCTATGTTCGATGCGCCTACTGCAGCACAGTTCTTGCGGTGGGAGTTCCATGCAAGAGGATG---
---ATGGACGCTGTGACTGTGAAATGTGGGCACTGCAACTCG---CTCTCTTACCTCGTGAAGCCTCCAGAG---
AAGAAGCACCGTCTTCCATCAGCCTATAATCGCTTCATGAGGGAGGAGATACAACGAATCAAGGCTGCAAATCCA
GAAATGCCACACCGCGAAGCTTTTAGCACTGCTGCAAAGAATTGGGCGAAGTATGAGCCTCGTGGACTAATTTGT
Asparagus_officinalis_CV289440
GAGCATGTCTGCTATGTCCACTGCAATTTCTGCAACACTATCCTTGCGGTCAATGTCCCCAGCAACAATTTGTTC
---AGCAGTATTGTGACAGTAAGGTGTGGACATTGCGCTAATCTTCTGTCAGTGAATATTCGCCCTCCTGAG---
AAGAGACAACGTGTTCCTTCAGCTTATAACAGATTTATCAAGGAAGAAATTCAAAGGATTAAGGCCAATAATCCA
GACATCAGTCATAGGGAAGCATTCAGTGCTGCCGCAAAGAATTGGGCACATTTTCCTCACATCCATTTCGGGCTC
Cabomba_caroliniana_AB553318
GAGCATCTCTGCTATGTGCGTTGCAACTTCTGCAGCACCGTTCTCGCAGTTGGTGTTCCCCGCAAGAGGCTG---
---CTTGATACAGTGACGGTGAAATGTGGTCACTGCAGCAAT---CTTTCTTTCATCGCAAAACCACCCGAG---
AAGAAGCACAGGCTTCCTTCAGCATACAATCGGTTCATGAGGGAGGAGATTCAACGACTCAAGGCAGCTGACCCG
AAGCTGACACACAAAGAAGCCTTCAGCAAGGCAGCTAAAAACTGGGCGCGGTTCGATCCTCAACTGCTAAATATT
Cabomba_caroliniana_AB553316
GAGCAGTTGTGCTACGTCCATTGCAACTTCTGTAATACGGTTCTAGCGGTGAGCGTTCCTTGCAGTAGCTTG---
---TTCAAGGTTGTGACTGTGAGGTGCGGCCATTGCACAAACCTCCTCTCTGTTAACAATAAGCCAACCGAG---
AAGAGGCAGAGAGTGCCATCGGCTTACAACAGGTTCATCAAAGACGAGATCCAACGAATCAAGGCAGGAAACCCA
GATATCACTCACAGAGAGACATTCAGCGCTGCTGCCAAGAATTGGGCACATTTCCCACATATTCACTTTGGTCTG
Cabomba_caroliniana_AB553317
GAGCAACTCTGCTATGTGCAATGTAGCTTCTGTGACACCATCTTGCTGGTAAGCGTGCCCTGCAGTAGCCTA---
---CTGAAAGTGGTGCCCATTCGATGCGGCCACTGTGGCAACCTTTTCTCCGTAAACAATAAACCTCCGGAG---
AAGAGACACAGAGCACCATCTGCTTACAATCGTTTCATAAAGGAAGAAATCCAAAGGCTCAAGGCCAATGACCCC
AACATCACTCACAGAGAGGCATTCAGCACAGCTGCTAAAAATTGGGCACACCTTCCTCGGTTTCAGCATAAGGCT
Cabomba_caroliniana_AB553319

CAGGTGAATGTGCCGTGCACTAACTCG------
CACAACATTGTAACTGTTCGATGCGGGCACTGTTCAGGTCTTCAATCAGTAAGCATCAAACCAACAGAA---
AAGAGGCAACGGGTTCCTTCAGCGTATAATCAATTCATCAAGGACGAGATCCAAAGGATAAAAGCTAGCAACCCA
GAGATCAGCCATAAGGAGGCATTCAGCACAGCTGCCAAAAATTGGGCTCATTTTCCTCACATTCAGTTTGGAATA
Cabomba_caroliniana_AB126655
GAGCAAGTGTGTTATGTCCACTGCAATTTTTGCAATACCATCCTTGCGGTGAGCGTTCCATGCACCAGCTTG---
---TTCAATATTGTGACTGTAAGATGTGGCCATTGCACCAATCTCTTGCCAGTAAACAATAGGCCTCCTGAA---
AAGAGGCAACGCGTCCCCTCGGCTTACAATCAGTTCATCAAGGAAGAGATACAAAGGATCAAGGCCAGTAACCCC
GAGATAAACCACAGAGAGGCGTTTAGTACTGCAGCAAAAAACTGGGCTCATTTTCCTCACATTCACTTTGGGCTG
Canna_indica_CRC
GAGCACCTTTGCTACGTGCGTTGCACGTACTGCAACGCTGTTCTTGCGGTTGGAGTGCCATGCAAGAGGGTG---
---ATGGACACTGTAACTGTGAAATGTGGCCACTGCAATCAC---CTCTCTTTTCTCGCCAAACCACCGGAG---
AAGAAACATAGAATGCCTTCTGCTTATAATCGATTCATGAAGGAGGAAATACAACGAATTAAAGCAGCTCAACCT
GACATCCCTCATCGAGAAGCTTTTAGCATGGCCGCGAAGAATTGGGCTAAGTGTGATCCTCGGTGCTCGACCATT
Canna_indica_2
GAGCGCATTTGCTATGTCCGCTGCAACTTCTGCAACACCATGCTTGCGGTGAGTGTCCCTGAGAACAGCTTAATG
---TTCAGTATTGTTACAGTGCGATGCGGGCATTGTTCTAATCTTCTATCAGTGAACATGCGTCCACCAGTG---
AAGAGACAACGTGTACCTTCGGCATATAACAAGTTTATCAAGGAGGAAATACAAAGGATAAAGGCTAATAATCCA
GACATCAGCCATAGAGAAGCATTTAGTACTGCCGCAAAGAATTGGGCCCATTTTCCTCACATCCAATTTGGTCTA
Canna_indica_1
GAGCAGGTTTGCTACATCCACTGCAACTTCTGCAACACAATCCTTGCGGTTAGTGTTCCTGGGCACAGCTTG---
---TTCAACATTGTGACAGTAAGATGTGGGCATTGTGCTAATCTGCTGTCTGTGAATTTGCGTCCCCCAGAG---
AAGAGGCAACGTGTTCCTTCTGCATATAACAGGTTTATCAGGGAGGAGATACGAAGGATAAAAGCTAACAACCCT
GATATTAGTCACAAGGAAGCATTCAGTGCTGCAGCAAAGAATTGGGCACACTTTCCTCATATACATTTTGGGCTA
Canna_indica_3 ---
TCTAGTGGTTAATATCCCGAGTGACAATTCA------
CTGAATGTTGTGACAGTAAGGTGTGGACTTTGTGCTAATTTACTGTCTGTGAATCCTGCAGCAACTGAG---
AAACGA---
CGTGTTCCTTCTGCCTACAACAGATTTATCAAGGAAGAGATACGTCGACTGAAGGCAAAAGATCCCAACATCAGC
CACAAGGAAGCCTTCAGCACTGCAG---
Citrus_clementina_CX308345
GAGCAACTCTGCTACATCCCCTGCAACTTTTGCAACATAGTTCTCGCGGTGAGTGTTCCTTGCAGTAGCTTG---
---CTTGACATCGTGACTGTCCGATGCGGGCACTGCTCCAATCTATGGTCCGTGAATAATCGTCCTCCCGAG---
AAGAGGCAGCGAGTACCTTCTGCATACAACCAGTTTATAAAAGAGGAGATTCAGAGGATCAAGGCTAATAATCCA
GATATCAGCCACAGGGAAGCATTCAGTACTGCCGCAAAGAATTGGGCACACTTCCCTCACATCCATTTTGGGCTG
Citrus_sinensis_CK936330
GAACGTGTTTGTTATGTTCACTGCAACTACTGCAACACCATTTTAGCGGTGAGTGTTCCTTGCTCAAGTTTG---
---TTCAACATTGTGACTGTTAGATGTGGGCATTGCGCCAATTTGCTAAATGTAAACATTCGTCCCCCAGAG---
AAGAGACAACGAGTTCCTTCTGCATACAATAGGTTTATCAAGGAGGAAATCCAAAGGATTAAGGCCAGTAACCCT
GACATCAGCCATAGAGAAGCTTTTAGCACAGCAGCAAAAAATTGGGCACATTTTCCTCACATTCACTTTGGATTA
Costus_pulverulentus_1
GAGAAGGTTTGCTATATCTTCTGCAACTTCTGCAATACAACCCTTGCGGTCAGTGTTCCTGGAATCAGCTTT---
---CTCAACATTGTGACTGTGAGATGCGGGCATTGTGCTAATCTGCTCACTGTGAACTTACGTCCCCCAGAG---
AAGAGGCAACGTGTTCCTTCAGCATATAACAGGTTTATCAGGGAGGAGATTCAGAGGATAAAGGCAAATAATCCA
GACATTAGTCATAAGGAAGCCTTCAGTGCTGCAGCGAAGAATTGGGCACACTTTCCTCACATTCATTTCGGGCTC

Costus_spicatus_3 --
CCTTGCGGTTAATGTTCCTGGAAACAGCTTG------
TTCAACATTGTCACAGTGAGATGCGGGCATTGTGCTAATCTGCTGTCCGTGAATTTACGTCCCCCAGAG---
AAGAGGCAACGTGTTCCTTCAGCATATAACAG--

Costus_spicatus_5 --
TCTCGTGGTTAATGTCCCAAGTAACAACTCG------
TCCAGCATTGTCGCAGTAAAATGTGGGCTTTGTGCTAATATACTGTCTGTGGACCTCATAAATCCTACA---
GAGAAGCGACGTGTTCCTTCTGCCTATAATAGATTTATAAAGGAAGAGATGCAGAGGCTTAAGGCAAAATATCCT
AATATTACCCACAAGCAAGCTTTTAGCATTGCAG--
Costus_spicatus_1

TCCAGAGGATAGATTAATG---
CTTAACATAATCACAGTTAGATGTGGCCACTGTGCAAATCTACTCCCGCTGCATATTCCACCCCCAGAA---
AAAAGACACCGAGTTCCTTCTGCATACAACAAGTTCATCAAAGAGGAGATTCAAAGGTTGAAGGCTAGTAATCCT
AGTATGAGTCACAAGCAAGCTTTCAGTAATGCAG--
Costus_spicatus_4 --
TCTAGCGGTTAGTGTGCCAGGAAATAGCTTG------
TTCAACATCGTGACAATACGATGTGGGCATTGCTCAAATCTGTTGTATGTGAACATGCGTCCCCCGGAG---
AAGAGG---
CGTGTTCCTTCTGCATATAACAGGTTCATAAAGGAGGAAATACAAA----------------------------
----
Costus_spicatus_2 --
CCTTGCGGTCAGTGTTCCAGGCAACAGACTG------
TTGAACTTGGTGACGGTACGCTGTGGGCATTGTGCAAATCTGCTCTCTGTGCACGTTCGCCCCCCTGAR---
AAGAGGCAACGTGTTCCATCTGCATATAATAGGTTTATCAAGGAAGAAATTCAAAGGATAAAGGCAAATAATCCG
AGCATTAGCCACAAGGAAGCATTCAGTGCAGCAG--
Costus_spicatus_CRC2

---GTGAAACCGCCTGAG---
AAGAAACACAGAATGCCTTCAGCATACAATCGCTTCATGAAGGAGGAAATACAGCGAATAAAAGCATCCAATCCT
GGTATTCCTCACCGGGAAGCATTTAGCATGGCTGCAAAGAATTGGGCAAAGTGTGATCCTCGTTGCTCAGTTATT
Costus_spicatus_CRC1
GAGCACCTCTGCTACGTGCGTTGCACCTACTGCAACACTGTTCTTGCGGTTGGAGTTCCATGCAAGAGGATG---
---ATTGACACATTAACTGTGAAATGTGGCCACTGC---AACCATCTCTCTTTTCTCGTCAAGCCTCCAGAA---
AAGAAACACAGAACCCCTTCAGCTTATAATCGCTTCATGAAAGAAGAAATAAAGCGAATCAAAGCAGCTCAACCT
ACAATTCCCCACCGAGAAGCTTTTAGCATGGCTGCAAAGAATTGGGCCAAGTGTGACCCTCGTAATTCAATTGTT
Cycas_rumphii_CB093468
GAACATTTGTGCTACGTGAGCTGCAGCTTTTGCAGCACCATTCTCGCGGTAAGCGTTCCATGCAGCAGCTTA---
---TTCAAGATCGTTACAGTGAGATGCGGTCATTGCACCAACCTTCTGTCGGTAAACAGACATGTGCCGGAG---
AAAAGGCAACGAGTTCCATCTGCATATAATCGCTTCATAAAAGAGGAGATTCAGAGGATCAAAGCCTGTCATCCT
GAGATAACTCACAGGGAAGCCTTCAGCACCGCTGCCAAAAACTGGGCACATTTTCCTCATATTCACTTCGGGTTG
Elaeis_guineensis_EL684075
GAGCAGCTCTGCTACGTCCACTGCAACTTTTGCGACACCGTCCTCGCGGTGAGTGTTCCCTGCAGCAGTTTG---
---TTCAAGACGGTGACGGTGAGGTGCGGCCACTGCACCAACCTCCTCTCCGTCAACAACAGACCTCCAGAG---
AAGAGGCAGAGAGTTCCCTCAGCATACAACCGATTTATCAAGGACGAAATCCAACGCATCAAAGCTGGGAATCCG

GACATCACTCACAGAGAGGCCTTCAGCGCCGCTGCTAAGAACTGGGCCCATTTCCCCCACATCCATTTCGGCCTG
Elaeis_guineensis_EL684122
GAGCAAGTCTGCTATGTCCACTGCAACTTCTGCAACACCATCCTGGTGGTTAGCGTCCCTGGCAGCAACTCC---
---CTCACCATTGTGACGGTAAGATGCGGGCACTGTGCTAATTTGCTGTCTGTGAATATTCACTTGCCAGAG---
AAGAGACAGCGTGTTCCTTCTGCTTACAACAGATTTATTAAAGAAGAAATACGAAGACTAAAGGCTACAAATCCT
GACATTAGCCACAGAGAAGCTTTCAGCGCTGCAGCAAAGAATTGGGCACACTTTCCTCATATCCAATTCGGGCTA
Eschscholzia_californica_HQ116795
GAGCAACTCTGTTACGTCCATTGCAACCTTTGTGACACCGTCCTCGCGGTAAGTGTTCCTTGCAGCAGTTTG---
---TTCAAGACGGTAACGGTTAGATGTGGACATTGCACAAACCTTCTGTCTGTGAACAACAGACCTCCGGAA---
AAGAGACAGAGAGTTCCCTCTGCTTACAATCGTTTCATCAAGGACGAAATCCAACGCATCAAAGCTGGAAATCCA
GATATTACCCATAGAGAAGCTTTCAGTGCTGCTGCAAAAAAATTGGGCTCACTTCCCACACATTCACTTTGGTCTT
Eschscholzia_californica_HQ116796
GAGCAACTTTGTTATGTTCATTGCAACCTTTGTGACACTGTCCTAGCAGTTAGTGTTCCTTGCAGCAGTTTG---
---TTTAAGACGGTAACGGTTCGATGTGGACATTGCACTAATCTTCTCTCTGTGAACAACAGACCCCCAGAA---
AAGAGACAGAGAGTCCCCTCTGCCTACAACCGTTTCATCAAGGAAGAAATCCAACGTATCAAAGCTGGAAATCCA
GATATTACCCATAGAGAGGCTTTCAGTGCAGCAGCCAAGAATTGGGCTCACTTCCCACACATCCACTTTGGTCTC
Eschscholzia_californica_HQ116797
GAACACGTTTGCTATGTTCACTGCAACTTCTGCAACACTATTTTAGCTGTTAGTGTACCAGGGACAAGTTTG---
---TTCAATGTTGTAACAGTTAGATGTGGTCATTGTGCTAATTTACTCTCTGTCAACCGCGCTGCCCCAGAG---
AAAAGGCAACGCGTTCCGTCTGCTTATAACCGATTTATCAAGGAAGAGATTCAAAGAATAAAGGCTAGTAATCCA
GATATTAGTCATAGGGAAGCTTTTAGCTCTGCAGCAAAAAAATTGGGCACATTTCCCTCACATTCACTTTGGACTA
Eschscholzia_californica_HQ116798
GAACACGTTTGTTATGTTCAGTGTAACTTCTGCAACACCATCTTAGCGGTGAGTGTACCAGGAACAAGTTTG---
---TTGAATGTGGTAACAGTTAGATGTGGTCATTGTGCTAATCTATTGTCTGTCAACGTACCTCCCCCGGAG---
AAGAGGCAACGTGTTCCATCCGCGTATAACCGATTTATCAAGGAGGAGATTCAAAGGATAAAAGCTACTAATCCA
GATATTAGTCATAGGGAAGCTTTTAGCACTGCAGCAAAAAATTGGGCTCATTTCCCTCACATTCACTTTGGATTA
Eschscholzia_californica_HQ116799
GAGCAAATCTGCTATATTCCTTGCAACTTCTGCAATATTGTTCTAGCGGTAAGTGTTCCATGCAGTAGCTTG---
---TTTGAGATTGTGACTATAAGATGTGGGCATTGCACTAATTTGTGGTCTGTAAACAATCGGCCTCCTGAA---
AAGAAGCATAGAGCACCATCTGCATATAATCAGTTCATCAAGGAGGAGATTCAAAGGATTAAGGCCAATCATCCA
AATATCACTCACAGAGAAGCATTCAGTACTGCAGCTAAGAATTGGGCACATTTTCCTCATACTCATTTTGGGTTG
Globba_laeta_3 --
CCTTGTGGTTTGTGTTCCAGGAAACGACTTGGCCAACAGCAGCATTGTGACAGTAAGATGTGGGCATTGCTCAAA
CCTTCTCTCTGTGAACCAGCCTCCACCTGAG---
AAGAGACAACGTGTTCCTTCTGTGTATAACAGGTTTATCAAGGAGGAAATACAAAGGATAAAGGCAAACAATCCA
GATATCAGT--
Globba_laeta_1

------------------GTACGCTGTGGGCATTGTGCAAATCTGCTCTCTGTGCACGTTCGYCCCCCTGGG---
AAGAGGCAACGTGTTCCATCTGCATATAATAGGTTTATCAAGGAAGAAATTCAAAGGRTAAAAGCAAATAATCCG
AGCATTAGCCACAAGGAAGCATTCAGTGCAGCAG---
Globba_laeta_4

CCGAGTGATAATTCA------
TTGAATGTGGCGACTGTCCGATGTGGTCTTTGTGCCAATCTACTCCCTATCAATCAACCGGCAACGGAT---
CAGAAGCGACGGGTTCCTTCTGCCTACAACAGATTTATCAAGGAAGAGATCCGACGCCTGAAAGCTAAAGATCCC

AACATCAGCCACAAGGAAGCTTTCAGCACTGCAG--
Globba_laeta_5

TTC------
ATTGATGTGGCGACTGTCCGATGTGGTCTTTGTGCCAATCTACTCCCTATCAATCAACCGGCAACGGAT---
CAGAAGCGACGGGTTCCTTCTGCCTACAACAGATTTATCAAGGAAGAGATCCGACGCCTGAAAGCTAAAGATCCC
AACATCAGCCACAAGGAAGCTTTCAGCACTGCAG--
Globba_laeta_2

---------ATTGTGACAGTGAGATGTGGGCGTTGTACTTGTCTGCTATCGGTAAACTTACATACCCCACAG---
AAAAGGCAGCGTGTTCCATCTGCCTACAACAAGTTTATCAGGGAGGAGATACAAAGGATAAAAGCCAATAACCCT
GATATCAGCCATAAGGAAGCTTTCAGTGCTGCGG--
Glycine_max_BU579031
GAACGTGTTTGTTATGTTCACTGCAACTTCTGCAACACCATTCTAGCGGTTAGTGTTCCATACAGCAGTTTG---
---CTAACCATAGTGACGGTTAGATGTGGACATTGTGCCAATTTGTTATCAGTTAACATTCGTCCTACAGAG---
AAGAGACATCGTGTTCCTTCTGCTTATAATCGCTTCATTAAGGAGGAAATTCAAAGGATTAAGGCTAGTAATCCA
GATATCAGCCACAGGGAGGCTTTCAGCTCAGCAGCCAAAAACTGGGCACATTTCCCTCACATTCACTTTGGGTTA
Glycine_max_CA800830
GACCAGCTCTGCTATGTCCATTGCAACTTCTGTGACACTGTCCTCGCGGTGAGTGTTCCTTGCACCAGCTTG---
---TTCAAGAATGTCACTGTGAGATGTGGTCATTGCACCAACCTTCTCTCAGTCAACAATAGACCTCCAGAG---
AAGAGACAGCGAGTTCCGTCTGCTTACAACCGCTTCATCAAGGATGAGATCCAACGTATCAAAGCTGGGAATCCT
GATATAAGCCACAGAGAGGCCTTTAGTGCAGCTGCAAAGAATTGGGCCCATTTTCCACACATTCATTTCGGACTC
Glycine_max_CA801979
GAGCAACTCTGCTACATCCCCTGCAACTTTTGCAATATTGTTCTTGCGGTGAGTGTTCCATGCAGTAGCCTG---
---TTTGACATTGTGACCGTTCGATGTGGGCACTGCACCAATCTATGGTCCGTGAACAACAGGCCTCCCGAG---
AAGAGGCAGCGCGTACCTTCTGCTTATAACCAGTTTATAAAGGAAGAGATTCAGAGGATCAAAGCCAATAATCCT
GATATCAGTCACAGAGAAGCTTTCAGTACAGCTGCAAAAAACTGGGCTCATTTTCCCCATATTCATTTCGGGCTG
Musa_acuminata_GSMUA_Achr11G03800
GAGCACCTCTGCTACGTCCACTGCAACTTTGCGACACCGACCTCGCGGTGAGTGTTCCCTACACCAGTCTC---
---TTCAAGACGGTCACCGTGAGGTGTGGTCACTGCACCAACCTTCTCTCGGTCAACAACAGACCTCCAGAG---
AAGCGGCAGAGAGTTCCGTCCGCGTACAACCGGTTCATCAAGGACGAAATCCAACGCATTAAGGCTGGGAATCCC
GACATCACGCACAGGGAAGCCTTCAGCGCCGCTGCAAAGAACTGGGCCCACTTTCCGCACATCCATTTCGGTCTG
Musa_acuminata_GSMUA_Achr11G17520
GAGCAGCTCTGCTACGTCCACTGCAACCTTTGCGACACCGTCCTCGCGGTGAGTGTTCCCTTTACCAGTTTG---
---TTCAAGACGGTGACTGTGAGGTGTGGCCACTGCACCAACCTTCTCTCCGTCAACAACAGACCTCCAGAG---
AAGAGGCAGAGAGTTCCTTCCGCATACAACCGGTTCATCAAGGACGAAATCCAACGCATTAAGGCTGGGAATCCC
GACATCACGCACAGAGAGGCCTTCAGCGCCGCTGCAAAGAACTGGGCTCACTTCCCACACATCCATTTTGGTCTG
Musa_acuminata_GSMUA_Achr1G04480
GAGCACTTGTGTTACGTGCGTTGCACCTACTGCAACACTGTTCTTGCGGTTGGAGTTCCAAGCAAGCCGATG---
---ATGGACACAGTAACAGTGAGATGTGGTCACTGCAACCAT---GTCTCCTTTCTTGTGAAACCTCCTGAG---
AAGAAACACAGAATGCCTTCAGCTTATAATCGCTTCATGAGGGAAGAAATACAGCGAATCAAAGCATCTAAACCC
GACATTCCTCACCGCGAAGCTTTTAGCATGGCTTCGAAGAATTGGGCAAAGTGTGATCCTCGTTGCTCAATTATC
Musa_acuminata_GSMUA_Achr1G24490
GAGCACATCTGCTACGTGCGTTGCAGCTATTGCAACACAGTTCTTAAGGTTGGCGTCCCATCCAAGAGGATG---
---ATAGACACAGTAACCGTGAGATGCGGTCACTGCGACCAT---CTCTCCTTCCTCACGAAACCTCCTGAG---
AAGAAACACAGAATGCCTTCAGCCTATAATCGCTTCATGAGAGAGGAAATACAGCGAATTAAAGCAGCAAAACCT

GATATTCCACACCGAGAAGCTTTCAGCAAGGCCTCGAAGAACTGGGCTAGGTGTGATCCTCGTCGGTCGACCTCG
Musa_acuminata_GSMUA_Achr1G27150
GATCGCCTGTGCTACGTGCGCTGCACCTACTGCAACACTGTTCTTGCGGTTGGCGTCCCTTGCAAGCGGGTG---
---ATGGACACGGTAACCGTGAAATGTGGTCACTGCAGCCAT---CTCTCCTTTCTGATGAAACCTCCGGAG---
AAGAAACACAGGATGCCTTCGGCTTACAATCGATTCATGAAGGAGGAAATACAACGAATCAAGGCAGCTAAACCT
GATATCCCGCACCGAGAAGCTTTTAGCATGGCTTCGAAGAACTGGGCCAAGTGTGATCCTCGTTGCTCAACTACT
Musa_acuminata_GSMUA_Achr3G25290
GAGCACCTTTGCTACATTCGTTGCACCTACTGCAACACTCTTCTTGCGGTTGGAGTTCCTCGCCGGTGGTTA---
---ATGGACAGCGTGACCGTGCATTGTGGTCACTGCAACCAT---CTATCCTTTCTCATCTGCAATTTACCA---
AAGAAACACAGAGCTCCATCAGCTTACAACCACTTCATGAGAGAGGAAATACAAAGAATCAAGGCAGCCAAACCT
GACATTCCACACCGCGAGGCATTTAGCATGGCTGCAAAGAATTGGGCTAAATGTGATCCTCGCAACTCAGCTAAT
Musa_acuminata_GSMUA_Achr3G25660
GAGCAAGCTTGCTACGTCAACTGCAACTTCTGCAACACTCCTCTCGTGGTTAATGTCCCAGGCAACAATTTA---
---CTCAATGTTGTCACAGTAAGATGTGGGCTCTGTGCTAATTTACTGTCTGTGGATATCCAACCAACCGAG---
AAGCGA---
CGTGTTCCATCTGCCTATAACAAGTTCATCAAGGAGGAGATACGGCGGCTGAAGGCAAAAGATCCCGACATCAGC
CACAGGGAAGCTTTCAGCACTGCAGCCAAAAACTGGGCACACTTCCCCGAAATCCATTTCGGGCCA
Musa_acuminata_GSMUA_Achr4G01430
GAGCACTTGTGCTACGTCCGCTGCACCTACTGCAACACAGTTCTTGCGGTTGGAGTTCCATGCAAGAGGCTG---
---ATGGACACAGTCACTGTGAAATGTGGTCACTGTAACCAT---CTCTCCTTCCTCGTCAAACCTCCCGAG---
AAGAAACACAGGATGCCTTCGGCCTATAATCGCTTCATGAAGGAGGAAATACAACGAATCAAAGCAGCTAAACCG
GATATTCCTCACCGAGAAGCTTTTAGCATGGCTGCTAAGAATTGGGCTAAATGTGATCCTCGTTGCTCAACCATC
Musa_acuminata_GSMUA_Achr4G22750
GAGCGCATTTGCTACGTCCACTGCAACTTCTGCAACACCGTCCTCGCGGTCAGTGTTCCTGGCAACAGCTCC---
---TTCAACAATGTAGCGGTACGATGCGGGCATTGTTCAATTCTGTTGTCTGTCAACACTCGACCCCCGGAG---
AAGAGACAACGCGTGCCATCTGCATATAACAAGTTTATCAAAGAGGAGATACAAAGGATAAAGGCTAAGAATCCA
GAGATCAGCCACAGAGAAGCATTCAGCACTGCAGCAAAAAATTGGGCACACTTCCCTCATATCCAGTTTGGGCTA
Musa_acuminata_GSMUA_Achr4G31310
AGTTATGTCCACTGCAACTTCTGCAACTTCTGCAACACCATCCTTGCGGTTAGTGTCCCCGGGAACAGCTTG---
---TTCAACATTGTCACAGTCCGATGCGGCCATTGCTCTAATCTGCTGTCTGTGGATATTCGCGCTCCCGGG---
AAGAGACAACGCGTACCTTCAGCGTATAACAAGTTTATCAAGGAGGAAATACAAAGGATAAAAGCTAATAATCCA
CACATTAGCCACAAACAAGCATTTAGCACTGCAGCGAAGAATTGGGCACACTTCCCTCACATCCATTTCGGGCAG
Musa_acuminata_GSMUA_Achr5G08930
GAGCACCTGTGTTACGTGCGTTGCACCTACTGCAACACTGTGCTTGCGGTTGGAGTCCCATGCAAGCCGGTG---
---ATGGACACGATCACCGTGAGATGTGGCCACTGCAACCAT---CTCTCCTTCCTCGTGAAACCTCCTGAG---
AAGAAACACAGAATGCCTTCAGCTTATAATCGCTTCATGAGGGAGGAGATTCAGCGAATCAAAGCTTCTAAACCC
GATATTCCTCACCGAGAAGCTTTTAGCATGGCTGCAAAGAACTGGGCAAAGTGTGATCCTCGTTGCTCGATCATT
Musa_acuminata_GSMUA_Achr6G24550
GAGCAACTGTGCTACGTGCGTTGCACCTACTGCAACACTGTTCTTGCGGTTGGAGTTCCATGCAAGCGGATG---
---ATGGATACAATTACTGTGAAATGTGGTCACTGCAACCAT---CTCTCCTTTCTCATGAAACCTCCCGAG---
AAGAAGCACAGAATGCCTTCTGCTTATAATCGCTTCATGAAGGAGGAAATACAACGAATCAAGGCAGCTAAACCT
GATATTCCTCACCGGGAAGCATTTAGCATGGCTGCAAAGAATTGGGCTAAGTGTGATCCTCGTTGCTCGTCGACT
Musa_acuminata_GSMUA_Achr6G31080
GAGCATATCTGCTACGTCCACTGCAACTTTTGCAACACGATCCTTGCGGTTAGTGTTCCAGGCAACAAACTG---
---TTCAACATGGTAACCGTTCGATGCGGACATTGCGCGAATTTGCTCTCTGTGCATGTTCGCCCCCCAGAA---
AAGAGACAACGTGTTCCTTCTGCTTACAACAGGTTTATCAAGGAGGAGATTCAAAGGATCAAGGCAAATAATCCT

AACATTAGCCACAAGGAAGCATTCAGTGCAGCAGCAAAGAATTGGGCACACTTTCCTCATATTCATATCGGGCGA
Musa_acuminata_GSMUA_Achr7G01330
GAGCACATTTGCTACGTCCACTGCAACTTCTGCAACACCATCCTTGCGGTGAGTGTCCCCGGCAACAGCTTG---
---TTCAGCATTGTAGCAGTGCGATGCGGGCATTGTTGTAATCTGCTGTCTGTGAACATGCGTCCTCCGGAG---
AAGAGACATCGCGTGCCGTCTGCATATAACAGGTTCATAAAGGAGGAAATACAAAGGATTAAGGCTAACAATCCA
GACATCAGCCACAGAGAAGCATTCAGCACCGCGGCGAAGAATTGGGCACACTTCCCTCACATCCATTTTGGACTC
Musa_acuminata_GSMUA_Achr7G07130
GAGCAGCTCTGCTACGCTTACTGCAACTTCTGCGACACCATTCTCGCGGTGAGCGTTCCCCGTAGTAGTCTT---
---TTCATGACGGTGACGGTGAAGTGCGGCAAGTGCACCAACCTTCTCTCTGTCAATAACAAACCTTTGGAG---
AAAAGGCAGAGAATTCCATCCGCATACAATCGATTTATCAAGGATGAAATCCAACGCATAAAAGCTGTGAATCCC
GACATTACTCACAGAGAGGCCTTCAGTGCAGCTGCAAAAAACTGGGCTCACTTTCCGCACATCCATTTCGGTCTG
Musa_acuminata_GSMUA_Achr7G07830
GAGCACGTTTGCTACATCCACTGCAACTTCTGCAACACCATCCTCGCGGTTAGCGTTCCCGGAAACAGCATG---
---TTCAACGTCGTGACAGTCCGGTGCGGGCATTGCGCCAATCTGCTGTCGGTCAACTTACGTCCCCCAGAG---
AAGAGGCAACGCGTTCCTTCCGCCTACAACAGGTTTATCAGGGAGGAGATACAAAGGATAAAAGCTAACAATCCT
GATATTAGCCACAAGGAAGCCTTCAGCGCTGCAGCGAAGAATTGGGCACACTTTCCTCACATTCATTTTGGGCTA
Musa_acuminata_GSMUA_Achr8G04340
GAGCACCTTTGCTACGTTCGTTGCACCTACTGCAACACTCTTCTTGCGGTTGGAGTTCCATTCAGGTGGTTG---
---ATGGACAGAGTGACTGTGAATTGTGGTCACTGCCACCAC---TTGTCCTTTCTCTTTGTTTTGTATGAT---
GTAAAACACAGAGCACCATCAGCTTACAATCACTTCATGAGAGAGGAAATACAGCGAATCAAGGCAGCCAAACCT
GACATACCTCATCGAGAAGCCTTCAGCATGGCAGCAAAGAATTGGGCTAATTCTGATCCGCGCAACTCATCTGAT
Musa_acuminata_GSMUA_Achr8G11580
GAGCAGCTCTGCTACGTCCACTGCAACTTCTGCGACACCGTCCTCGCCGTGAGTGTTCCCTACACCAGTCTC---
---TTCAAGACGGTGACGGTGAGGTGTGGCCACTGCACCAATCTTCTCTCCGTCAACAACAGACCTCCGGAG---
AAGCGGCAGCGAGTTCCATCCGCATACAATCGGTTCATCAAAGACGAAATCCAACGCATTAAAGCTGGGAATCCC
GACATCACTCACAGGGAGGCCTTCAGCGCCGCTGCAAAGAACTGGGCCCACTTTCCGCACATCCATTTCGGACTG
Heliconia_caribaeaxbihai_1

------AACATTGTAACAATACGATGTGGGCATTGTGCTAATCTGCTGTCTGTCAACTTACGTCCCCCAGAG---
AAGAGGCAACGCGTTCCTTCTGCATATAACAGGTTTATCAGGGAGGAGATTCAAAGGATAAAAGCTAACAATCCT
GATATTAGCCACAAGGAAGCTTTCAGTGCTGCAG--
Heliconia_caribaeaxbihai_2

CTTGCGGTTAGTGTCCCTGGCAACAGCTTG------
TTCAACATTGTAACAGTGCGATGCGGGCATTGTTCCAATCTGCTGTCTGTGAATATTCGTCCCCCAGAG---
AAGAGACATCGTGTGCCTTCTGCGTATAACAGGTTCATCAAGGAGGAAATACAAAGGATAAAGGCCAACAATCCA
GACATCAGC---
Heliconia_caribaeaxbihai_3

CCGAGTGATAATTCATTG---------
GATGTGGCGACTGTCCGATGTGGTCTCTGTGCCAATCTGCTCACTGTCAATCAACCCGCAACGGAG---
AAGCGA---
CGTGTTCCTTCTGCCTACAACAGATTTATCAAGGAAGAGATCAGACGACTGAAAGCTAAAGATCCCAACATTAGC
CACAAGGAAGCTTTCAGCACTGCAG--
Heliconia_pendula_2 --
CCTTGCGGTCAGTGTTCCTGGAAACAGCTTG------

TTCAACATTGTAACAATACGATGTGGGCATTGTGCTAATCTGCTGTCTGTCAACTTACGTCCCCCAGAG---
AAGAGGCAACGCGTTCCTTCTGCATATAACAGGTTTATCAGGGAGGAGATTCAAAGGATAAAAGCTAACAATCCT
GATATTAGCCACAAGGAAGCTTTCAGTGCTGCAG---
Heliconia_pendula_1 ---
CCTTGCGGTTAGTGTTCCAGGCAACAGACTA------
TTCAACATAGTAACTGTACGATGTGGCCATTGTGCAAATCTACTCTCTGTACACGTTCGCCCCCCAGAA---
AAGAGACAACGCGTTCCTTCTGCATATAACAGGTTCATCAAGGAGGAGATTCAAAGGATAAAGGCAAATAATCCT
AACATTAGCCATAAGGAAGCATTCAGCGCAGCTG---
Heliconia_pendula_3

GCCAATTTG------
TTCAACAATGTGACAGTAAGATGTGAGCATTGCTCCAATTTTCTGTCTGTGAATATTCGCCCAGTGGAG---
AAGAGACAACGCATTCCTTCTGCCTATAACAGATTTATTAAGGAGGAGATAAGACGACTAAAAGCGACAAATCCA
GACATTAGCCATAGGGAAGCTTTCGGCACTGCAG---
Hordeum_vulgare_AK250112
GAACAAGTCTGCTACGTCCACTGCAACTTCTGCAACACCGTACTCGCGGTGAGTGTCCCTGGGAACAGCATG---
---TTCAACGTCGTGACGGTCCGGTGTGGGCACTGCACGAACCTGCTTTCGGTGAGCGCACGCCCTCCGGAG---
AAGAGGCAGCGCGTTCCTTCTGCGTACAACAGATTCATCAAGGAAGAGATACGAAGGATCAAAGCAAACAACCCC
GACATTAGCCACAGGGAAGCCTTCAGCACTGCCGCAAAGAACTGGGCACATTACCCAAACATCCACTTCGGGCTA
Lilium_longiflorum_EF363135
GACCACCTCTGCTACGTTCGTTGCACCTACTGCAACACTGTTCTTGCGGTAGGAGTACCATGCAAGCGATTG---
---ATGGATACGGTGACAGTGAAGTGTGGTCACTGCAACCATATCTCCTTCATCAACGTCAAGCCACCTGAG---
AAGAAACACAGGCTTCCATCTGCTTACAATCGCTTCATGAAGGAGGAAATACAGCGAATCAAAGCTGCCAAGCCT
GATATTCCTCATCGAGAAGCTTTTAGCATGGCTGCAAAGAACTGGGCTAAGTGTGACCCTCGGGCAACAAATACT
Marantochloa_leucantha_1 ---
CCTTGCGGTGAGTGTTCCAGGGAGCAGGCTC------
TTCAACATAGTAACTGTGAGATGCGGCCATTGTGCAAATCTGCTCACTGTGCACGTTCGCCCCCCAGAA---
AAGAGACAGAGAGTTCCTTCTGCATATAACAGGTTTATAAGAGAGGAGATTCAAAGGATAAAGACAAGCAACCCC
AATATCACCCACAAGGAAGCATTCAGTACAGCAG---
Marantochloa_leucantha_2 ---
CCTTGCGGTCAGTGTTCCAGGCAACAGACTG------
TTGAACTTGGTGACGGTACGCTGTGGGCATTGTGCAAATCTGCTCTCTGTGCACGTTCGCCCCCCTGAG---
AAGAGGCAACGTGTTCCATCTGCATATAATAGGTTTATCAAGGAAGAAATTCAAAGGATAAAAGCAAATAATCCG
AGCATTAGCCACAAGGAAGCATTCAGTGCAGCAG---
Marantochloa_leucantha_3

GATAAATTG------
TTCGACACTGTGACAGTAAGATGTGAGCATTGCTCCAATTTGCTGTCTGTGAATATTCACCCAGTTGAG---
AAGAGACAACGCATTCCTTCTGCCTATAACAGATTTATTAAGGAGGAGATAAGACGATTAAAAGCCACGAATCCA
GACATCAGCCACAAGGAAGCTTTCAGCACTGCAG---
Musa_acuminata_1 ---
CCTCGCGGTCAGTGTTCCTGGCAACAGCTCC------
TTCAACAATGTAGCGGTACGATGCGGGCATTGTTCAAATCTGTTGTCTGTCAACACGCGACCCCCAGAG---
AAGAGACAACGCGTGCCTTCTGCATATAACAAGTTTATCAAAGAGGAGATACAAAGGATAAAGGCTAAGAATCCA
GAGATCAGCCACAGAGAAGCATTCAGCACTGCAG---
Musa_acuminata_2

GAGCACATTTGCTACGTCCACTGCAACTTCTGCAACACCATCCTTGCGGTGAGTGTCCCCGGCAACAGCTTG---
---TTCGGCATTGTAGCAGTGCGATGCGGGCATTGTTGTAATCTGCAGTCTGTGAACATACGTCCTCCAGAG---
AAGAGACATCGCGTGCCGTCTGCGTATAACAGGTTCATAAAGGAGGAAATACAAAGGATTAAGGCTAACAATCCA
GGCATCAGCCACAGAGAAGCATTCAGCACCGCGGCAAAGAACTGGGCACACTTCCCTCACATCCATTTTGGACTC
Musa_basjoo_2 --
CCTCGCGGTTAGCGTTCCCGGAAACAGCATG------
TTCAACCTCGTGACAGTCCGGTGCGGCATTGCGCCAATCTGCTGTCGGTCAACTTACGTCCCCCAGAG---
AAGAGGCAACGCGTTCCTTCTGCCTACAACAGGTTTATCAGGGAGGAGATACAAAGGATAAAAGCTAACAATCCT
GATATTAGCCACAAGGAAGCCT--
Musa_basjoo_6

--TCCGTGAATCAA---CCAACAGAG---
AAGCGT---
CGTGTTCCTTCTGCCTATAACAGGTTCATCAAAGAGGAGATACGTCGGCTGAAGACGAAAAATCCCAACATTAGC
CACAAGGAAGCTTTCAGCACCGCA--
Musa_basjoo_3 --------------------------
CTTCTGCAACACCATCCTTGCGGTTAGTGTCCCCGGGAACAGCTTG------
TTCAACATTGTCACAGTCCGATGCGGCCATTGCTCTAATCTGCTGTCTGTGGATATTCGCGCTCCCGGG---
AAGAGACAACGCGTACCTTCAGCGTATAACAGGTTTATCAAGGAGGAAATACAAAGGATAAAAGCTAATAATCCA
CACATTAGCCACAAACAAGCATTTAGCACTGCGGCGAAGAATTGGGCACACTTCCCTCACATCCATTTCGGGCTG
Musa_basjoo_5
GAGCACATTTGCTACGTCCACTGCAACTTCTGCAACACCATCCTTGCGGTGAGTGTCCCCGGCAACAGCTTG---
---TTCGGCATTGTAGCAGTTCGATGCGGGCATTGTTGTAATCTGCTGTCTGTGAACATACGTCCTCCGGAG---
AAGAGACATCGCGTGCCGTCTGCGTATAACAGGTTCATAAAGGAGGAAATACAAAGGATTAAGGCTAACAATCCA
GACATCAGCCACAGAGAAGCATTCAGCACYGCRGCAAARAAYTGGGCACACTTCCCTCAYATCCAKTTTGGRCTM
Musa_basjoo_4
GAGCACATTTGCTACGTCCACTGCAACTTCTGCAACACCGTCCTCGCGGTCAGTGTTCCTGGCAACAGCTCC---
---TTCAACAATGTAGCGGTACGATGCGGGCATTGTTCAAATCTGTTGTCTGTCAACACGCGACCCCCAGAG---
AAGAGACAACGCGTGCCTTCTGCATATAACAAGTTTATCAAAGAGGAGATACAAAGGATAAAGGCTAAGAATCCA
GATATCAGCCACAGAGAAGCATTCAGCACTGCAGCAAAA------------------------------------
Musa_basjoo_1
GAGCATATCTGCTACGTCCACTGCAACTTTTGCAACACGATCCTTGCGGTTAGTGTTCCAGGCAACAAACTG---
---TTCAACATGGTAACCGTTCGATGCGGACATTGCGCGAATTTGCTCTCTGTGCATATTCGCCCCCCAGAA---
AAGAGACAACGTGTTCCTTCTGCTTACAACAGGTTTATCAAGGAGGAGATTCAAAGGATCAAGGCAAATAATCCT
AGCATTAGCCACAAGGAAGCATTCAGTGCAGCAGCAAAGAATTGGGCACACTTT---------------------
Nymphaea_alba_AB092980
GAGCAACTCTGCTATGTGCAGTGCAGTTTCTGTGATACCATCTTGCTGGTAAGTGTCCCTTGCAGTAGCTTG---
---CTGAAAGTGGTGCCTGTCAGATGTGGCCATTGTAGCAACCTTTTTTCGGTAAACAATAAGCCTCCAGAG---
AAGAGACACAGAGCTCCTTCTGCTTACAACCGTTTCATAAAGGAAGAGATCCAGAGGCTTAAGACCAGTGAGCCA
AACATCAGCCACAGGGAGGCATTCAGCACTGCTGCTAAAAATTGGGCACACATGCCTAGAATTCAGCATAAACCA
Nymphaea_colorata_AB092981
GAGCAACTCTGCTATGTGCAGTGCAGTTTCTGTGATACCATCTTGCTGGTAAGTGTCCCTTGCAGTAGCTTG---
---CTGAAAGTGGTGCCTGTCAGATGTGGCCATTGTAGCAACCTCTTTTCAGTCAACAATAAACCTCCAGAG---
AAGAGACACAGAGCTCCTTCTGCTTACAACCGGTTCATAAAGGAAGAGATCCAGAGGCTCAAGACCAGTGAGCCA
AGCATCAGCCACAGGGAGGCACTCAGCACTGCCGCTAAAAATTGGGCACATTTACCTCGAATTCAGCATAAACCA
Orchidantha_siamensis_3 --

CCTCGCGGTTAGTGTCCCTGGCGACAGCTTG------
TTCAACATTGTAACAGTACGATGCGGTCATTGTTCTAATCTTCTTTCTGTGAACATGCGTCCCCCCAAG---
AAGCGACAACGTGAACCTTCTGCATATAACAGGTTTATCAAGGAGGAAATACAAAGGATAAAGGCTAACAATCCG
GACCTCAGCCACAGAGAAGCATTCAGCACTGCAG---
Orchidantha_siamensis_1 ---
CCTCGCGGTTAGTGTCCCWGGCRACAGMWTG------
TTCAACATWGTRACWGTACGATGCGGKCATTGTACAAATCTACTCTCTGTACATGTTCGCCCCCCAGAA---
AAGAGACAACGCGTTCCTTCTGCTTATAACAGGTTTATCAGGGAGGAGATACAAAGGTTAAAGGCTAATAATCCT
AACATTAGCCACAAGGAAGCATTCAGTGCAGCAG---
Orchidantha_siamensis_2 ---
TCTTGCGGTTAGTGTTCCCGGAAATTGCTTG------
TTCAACATTGTGACAATAAGATGCGGGCATTGTGCTAATCTGCAGTCCGTGAACTTACGTCCCCCGGAGACGAAG
AGGCAGCGCGTTCCTTCTGCATACAACAGGTTTATCAGAGAGGAGATCCAAAGGATAAAGCTAACAATCCTGAT
ATTAGCCATAAGGAAGCTTTCAGTGCTGCGG---
Orchidantha_siamensis_4

---TCGGTGAATATCCAACCAACGGAG---
AAGCGA---
CGTGTTCCTTCTGCCTATAACAGATTTATCAAGGAGGAGATCCGGCGACTAAAGGAAACAAATCCCAACATTAGC
CACAAGGAAGCATTCAGCACAGCAG---
Oryza_sativa_AB106553
GAGCACCTGTGCTACGTGCGCTGCACCTACTGCAACACCGTGCTCGCGGTTGGAGTCCCATGCAAGAGGCTG---
---ATGGACACCGTGACCGTGAAATGTGGCCACTGCAACAAC---CTCTCCTTCCTCGTGAAGCCCCCAGAG---
AAGAAACACCGCCTCCCATCTGCTTACAACCGCTTCATGAGGGAGGAAATACAGCGTATCAAAGCTGCCAAGCCA
GATATCCCTCACAGGGAGGCCTTCAGCATGGCTGCCAAGAACTGGGCGAAGTGCGACCCCCGCTGCTCATCGACG
Oryza_sativa_AB274013
GAGCATGTTTGCTATGTCAACTGCAACTATTGCAACACTATCCTTGTGGTGAATGTGCCAAACAATTGTTCC---
---TACAACATTGTGACTGTTAGATGTGGGCATTGCACAATGGTGCTCTCCATGGATATACGTCCCCCTGAG---
AAAAGACAGCGTGTCCCATCTGCATACAACAGATTCATCAAGGAGGAGATACAGAGGATTAAAACCAGCAATCCT
GAGATTAGCCACAGGGAGGCATTCAGTGCTGCTGCAAAGAACTGGGCTCATCTTCCCCGGCTCCATTTTGGCCTC
Oryza_sativa_AB274014
GAGCATGTGTGCTACGTGCACTGCAACTTCTGCAACACAATTTTCGCGGTCAGTGTTCCAAGCAATAGCATG---
---CTAAACATCGTGACCGTCCGTTGTGGCCATTGCACTAGCCTGTTGTCAGTGAACGTGCGACCTCCAGAG---
AAGAGGCAGCGGGTTCCTTCAGCTTATAACAGATTTATCAAGGAAGAGATCCGGAGGATAAAAGCAAACAATCCT
GACATAAGCCACAGAGAAGCCTTCAGTACTGCAGCAAAGAATTGGGCGCATTTCCCGAACATCCATTTCGGGTTA
Oryza_sativa_AB274015
GAGCAGCTCTGCTACGTGCACTGCCACTACTGCGACACCGTGCTCGTCGTGAGCGTGCCGAGCAGCAGCCTG---
---TTCGAGACGGTGACGGTGAGGTGCGGCCACTGCAGCAGCCTCCTCACCGTCAACAACAGACCTCCGGAG---
AAGAGGCAGCGTGTGCCATCGGCGTACAACCGCTTCATCAAGGACGAAATCCAACGCATCAAGGCTGGCAATCCC
GACATCTCGCACAGGGAGGCCTTCAGCGCGGCTGCCAAGAACTGGGCACACTTTCCGCACATCCACTTTGGACTG
Oryza_sativa_AB274016
GAGCAGCTCTGCTACGTGCACTGCAACTGCTGCGACACCATCCTCGCCGTCGGCGTGCCGTGCAGCAGCCTG---
---TTCAAGACGGTGACGGTGCGGTGCGGCCACTGCGCCAACCTGCTCTCCGTCAACAACAGGCCTCCGGAG---
AAGCGTCAGAGAGTCCCATCTGCGTACAACCGGTTCATCAAGGATGAGATCCAGCGGATCAAGGCCGGCAACCCG
GACATCAGCCACCGGGAGGCGTTCAGTGCAGCCGCAAAGAATTGGGCCCATTTCCCTCACATCCATTTTGGCCTC
Oryza_sativa_AB274017

GAGCAGCTCTGCTACGTGCACTGCAACTTCTGCGACACCATCCTCGCGGTGGGCGTGCCGTGTAGCAGCCTG---
---TTCAAGACGGTCACCGTGCGGTGCGGCCACTGCGCCAACCTGCTCTCCGTCAACAACAGGACTTCAGAG---
AAGCGGCAGAGGGTTCCCTCGGCATACAACCGATTCATCAAAGATGAGATACAACGCATCAAGGCAAGCAATCCG
GACATCACCCACAGGGAGGCTTTCAGCGCTGCTGCCAAGAACTGGGCCCATTTCCCACACATCCATTTTGGCCTG
Oryza_sativa_AB274018
GAGCAAGTGTGCTATGTGCACTGCAACTTCTGCAACACGATTCTCGCGGTGAGTGTCCCAGGAAACAGCATG---
---CTGAACATCGTGACCGTCCGGTGTGGGCACTGCACGAATCTGCTGTCAGTGAACGCTCGCCCTCCAGAG---
AAGAGGCAAAGGGTACCTTCAGCCTATAACAGATTTATTAAGGAAGAGATACGAAGGATCAAAGCAAACAACCCC
GACATTAGCCACAGGGAAGCCTTCAGCACAGCAGCCAAGAATTGGGCACATTATCCAAACATCCATTTTGGCCTA
Oryza_sativa_AB274019
GAGCGGCTAGGCTGCGTGCAGTGCAGCTTCTGCGCCACCGTGTTGCTGGTGAGCGTGCCGTGCAGCAGCGTG---
---CTCAGGGTGGTGGCCGTGCAGTGCGGCCACTGCTCCGGCATCCTCTCCGCCGTCAACAAGCCGCCAGGG---
AGGAAGCAGCGGACGCCGTCTGCCTACAACTGTTTCGTCAAGGAAGAGATAAAGAGGATCAAAAGCATGGAGCCT
AACATCACCCACAAACAAGCATTCAGCACAGCTGCTAAAAACTGGGCTCACTTACCCAGAATCCAGCAGAAGCGA
Phyllostachys_edulis_FP099409
GATCAGCTCTGCTACGTGCACTGCAACTTCTGCGACACCATCCTCGCGGTGGGCGTGCCGTGCAGCAGCATG---
---TTCAAGACGGTCACCGTGCGGTGCGGCCACTGCGCCAACCTGCTATCCGTCAACACCAGGCCTGCAGAG---
AAGCGGCAGAGAGTTCCCTCGGCTTACAACCGATTCATCAAAGACGAGATACAGCGCATCAAGGCCAGCAACCCG
GACATCACCCACAGGGAGGCTTTCAGCGCAGCTGCCAAGAATTGGGCTCATTTCCCGCACATTCATTTCGGTCTG
Picea_glauca_BT115385
GATCACCTCTGCTACGTTCACTGCGACTTTTGTAGCACAATCCTCGCGGTAAACGTTCCATGCAACAGCTTA---
---TACAGGATTGTTACAGTCCGATGCGGTCATTGCACTAACCTCTTGTCGGTAAACTACAGACAGCCCGAG---
AAGAAACAGCGCGTTCCATCTGCATATAATCGTTTCATTAGAGACGAAATCCAGAGGATCAAAGCCAACAATCCA
AAAATAACTCACAAGGAAGCTTTTAGCGCCGCTGCCAAGAACTGGGCACATTATCCTCATATTCACTTCGGGTTG
Picea_sitchensis_BT123811
GGGCATTTGTGCTATATTCAGTGCAATTATTGCAGCACTGTCCTAGCGGTGAATGTCCCGGGCAGTAGTTTA---
---TTGGAGATTGTCCCAGTGCGGTGCGGTCATTGCACCAGCCTTCTATCAGTTAACTGCGGGACTACAGAA---
AAAAGACAACGAGCTCCATCCGCCTATAACCGATTCATAAGAGCGGAAATACAGAGGATCAAAGCCGTCAATCCG
GAGATCAGCCATAGGGAAGCTTTCAGCGCTGCTGCCAAGAACTGGGCTCACTTAGGGTTGATGCTGCCGGATAAC
Picea_sitchensis_BT124509
GAGCAGCTGTGCTACGTGCACTGTAACTTTTGCAGCACCATTCTAGCGGTGAGCGTTCCGTCCAGTAGCTTA---
---TTCAATATAGTCACAGTTCGCTGTGGGCATTGCAACCATCTCCTATCAGTTAACAGCATACCACCTGAA---
AAGAGACAACGAGTACCGTCCGTGTACAATCGTTTCATAAAAGAGGAAATTCAAAGAATCAAAGCCAAAAATCCG
GACATAAGGCACAAGGAGGCTTTCAGTGCCGCTGCAAAAAATTGGGCTCATTTTCCTCATATACATTTCGGATTG
Picea_sitchensis_EF086340
GATCACCTCTGCTACGTTCACTGCGACTTTTGTAGCACAATCCTCGCGGTAAACGTTCCATGCAACAGCTTA---
---TACAGGATTGTTACAGTCCGATGCGGTCATTGCACTAACCTTTTGTCGGTAAACTACAGACAGCCCGAG---
AAGAAACAGCGCGTTCCATCTGCATATAATCGTTTCATTAGAGACGAAATCCAGAGGATCAAAGCCAACAATCCA
AAAATAACTCACAAGGAAGCTTTTAGCGCCGCTGCCAAGAACTGGGCACATTATCCTCATATTCACTTCGGGTTG
Pinus_taeda_DR100835
GATCACCTCTGCTACGTTCACTGCGACTATTGCAACACAATCCTCGCGGTAAACGTTCCATGCAACAGCTTA---
---TACAGGACTGTTACAGTCCGATGCGGTCATTGCAATAACCTCCTGTCGGTAAACTACAGACAACCCGAG---
AAAAAACAGCGCGTTCCATCTGCATATAATCGTTTCATTAGAGATGAAATCCAGAGGATCAAAGCCCACAATCCA
AAAATAACTCACAAGGAAGCTTTCAGCGCCGCTGCCAAGAACTGGGCACATTATCCTCATATTCACTTTGGGATG
Ruscus_aculeatus_AB168115
GAGCATGTCTGTTATGTCCACTGCAACTTCTGCAACACCATCCTCGTGGTTAATGTCCCTGGAAACAATTTG---

---TTCAACATTGTGACCATAAGGTGTGGGCATTGTGCTAATCTTCTATCAGTGAATATTCGCTCTCCTGAG---
AAGAGACAACGTGTTCCCTCAGCTTATAATAGATTTATCAAGGAGGAGATTCAAAGAATCAAGGCCAACAATCCT
GACATTAGCCATAGGGAAGCATTCAGTGCTGCAGCAAAGAATTGGGCACATTTTCCACACATCCATTTCGGGCTC
Saccharum_officinarum_CA277458
GAGCATGTGTGCTACGTGCACTGCAACTTCTGCAACACAATTCTCGCGGTCAGTGTCCCGAGTCACAGCATG---
---CTGAACATCGTGACAGTCCGTTGTGGGCACTGCACTAGCCTGCTGTCAGTGAACGTGCGTGCTCCAGAG---
AAGAGGCAACGCGTTCCTTCAGCATATAACAGATTTATTAAGGAAGAGATACGAAGGATTAAAGCAAGCAACCCA
GACATAAGCCATAGGGAAGCCTTCAGCACTGCAGCGAAGAATTGGGCACATTTTCCAAACATTCATTTTGGACTA
Schumannianthus_virgatus_1 --
CCTTGCGGTCAGTGTTCCAGGCAACAGACTG------
TTGAACTTGGTGACGGTACGCTGTGGGCATTGTGCAAATCTGCTCTCTGTGCACGTTCGCCCCCCTGAA---
AAGAGGCAACGTGTTCCATCTGCATATAATAGGTTTATCAAGGAAGAAATTCAAAGGATAAAGGCAAATAATCCG
AGCATTAGCCACAAGGAAGCATTCAGTGCAGCAG--
Schumannianthus_virgatus_3 --
GCTTGCGGTTAATGTCCCTGGAAACAATTTG------
TTGAAGATTGTGGCTGTAAGATGTGGTCATTGTTCTAATCTGCTTTCTGTGCATACACCTTCTCCAGAG---
AAGAAACAACGCGTGCCTTCTGCGTATAACAGGTTTATCAAAGAGGAAATACAAAGGATAAAAGCTAATAATCCA
GAAATTAGCCACAGAAAAGCATTCAGCACTGCAG--
Schumannianthus_virgatus_2 --
CCTAGCGGTTGGYGTTCCTGGAAGCAGCTTG------
TTCAACATTGTGACTGTGAGATGTGGGCACTGCGCTAATCTGCTGTCTGTGAACTTGCGCCCCCCAGAG---
AAGAGGCAACGTGTTCCATCTGCATATAACAGGTTTATCAGGGAGGAGATACAAAGGATAAAAGCGAACAATCCT
GATATCAGTCACAAGGAAGCTTTCAGTGCTGCAG--
Sorghum_bicolor_XM002452807
GAGCAGCTCTGCTACGTGCACTGCAACTGCTGCGACACCATCCTCGCCGTCGGCGTGCCTTGCAGCAGCCTG---
---TTCAAGACGGTGACGGTGCGCTGCGGCCACTGCGCCAACCTACTCTCCGTCAACAACAGGCCTCCGGAG---
AAGCGGCAGAGAGTCCCGTCGGCATACAACCGGTTCATCAAGGACGAGATCCAGCGCATCAAGGCCGGGAACCCG
GACATCACCCACCGGGAGGCGTTCAGCGCAGCCGCAAAGAATTGGGCCCATTTCCCACACATCCACTTCGGCCTC
Sorghum_bicolor_XM002448190
GAGCAGATCTGCTACGTGCACTGCAGCTACTGCGACACCATCCTCGCGGTGGGCGTGCCGTGCAGCAGCTTG---
---TTCCAGACGGTCACCGTGCGGTGCGGCCACTGCTCCAACCTGCTCTACGTCAACAACAAAACTTCAGAG---
AAGCGGCAGAGAGTTCCTTCCGCGTACAACCGTTTCATCAAAGATGAGATCCAGCGCATCAAGGCCAGCAATCCG
GACATCACTCACAGGGAAGCTTTCAGCGCGGCTGCCAAGAATTGGGCCCATTTCCCGCACATCCATTTCGGTCTC
Sorghum_bicolor_XM002459322
GAGCATGCCTGCTATGTCAATTGCAACTATTGCAACACTATCCTGGTGGTGAATGTGCCAAACAGTTGTTCC---
---CACAACATTGTGACTGTCAAATGTGGGCATTGTACAATGGTGCTCTCTATGGATATAAGACCCCCAGAG---
AAGAGGCAACGTGTCCCATCAGCATACAACAGATTTATCAAGGAAGAAATACAAAGGATAAAGACGAGCAACCCA
GAGATTAGCCACAGGGAAGCATTCAGTGCTGCAGCGAAGAATTGGGCTCATCTTCCTCGGCTCCATTTCGGCCTC
Sorghum_bicolor_XM002464354
GAGCAGCTCTGCTACGTGCACTGCCACTTCTGCGACACCGTCCTCGTCGTGAGCGTGCCTACGAGCAGCTTG---
---TTCAAGACGGTGACGGTACGATGCGGCCACTGCAGCAGCTTGCTCACTGTCAACAACAGACCTCCGGAG---
AAGAGGCAGCGCGTGCCGTCGGCGTACAACCGCTTCATCAAGGACGAAATCCAACGCATCAAGGCTGGCAATCCC
GACATCTCGCACAGGGAGGCCTTCAGCGCGGCCGCCAAGAACTGGGCGCACTTTCCACACATCCACTTTGGACTC
Strelitzia_sp_5 --
CCTCACGGTTAACATTCCATGCGATAAATTG------
TTCGACACTGTGACAGTAAGATGTGAGCATTGCTCCAATTTGCTGTCTGTGAATATTCGCCCAGTTGAG---

AAGAGACAACGCATTCCTTCTGCCTATAACAGATTTATTAAGGAGGAGATAAGACGATTAAAAGCCACGAATCCA
GACATCAGCCACAAGGAAGCTTTCAGCACTGCAG---
Strelitzia_sp_3   ---
CCTTGCGGTCAGTGTCCCTGGCAACAGCTTG------
TTCAACATTGTGACAGTTCGATGCGGGCATTGTTCTAATCTGCTTTCTGTGAACATGCGTCCGCCGGAG---
AAGAGACAACGCGTACCTTCTGCATATAACAGGTTTATCAAGGAGGAAATACAAAGGATAAAGGCTAACAATCCA
GACATCAGCCACAGAGAAGCCTTCAGCACTGCAG---
Strelitzia_sp_2   ---
CCTTGCGGTTAGTGTTCCTGGAAACAGCTTG------
TTCAACATTGTGACTGTACGGTGCGGGCATYGTGCTAATCTGCTGTCTGTGAACTTACGTCCCCCAGAG---
AAGAGGCAACGTGTTCCTTCTGCATATAACAGGTTTATCAGGGAGGAGATACAAA--------------------
----
Strelitzia_sp_4

CCCTGGCAACATCTTG------
TTCAACATTGTAACAGTACGATGCGGGCATTGTTCTAATCTGCTGTCTGTGAATATTCATCACCCTGAG---
AAGAGACAACGCGTGCCTTCTGCATATAACAGGTTTATCAAGGAGGAAATACAAAGGATAAAGGCAAACAATCCA
AACATCAGCCACAGAGAAGCATTCAGCACTGCAG---
Strelitzia_sp_1   ---
CCTCGCGGTTAGTGTCCCAGGCAACAGATTG------
TTCAACATAGTAACTGTACGATGTGGGCATTGCGCAAATCTACTCTTTGTACATATTCGCCCCCCAGAA---
AAGAGACAACGTGTTCCTTCTGCTTATAACAGGTTTATCAAGGAGGAGATTCAAAGGTTAAAGGCTAATAATCCT
AA---
Triticum_aestivum_BT009106
GAAGAAGTCTGCTACGTGCACTGCAACTTCTGCAACACCGTACTCGCGGTGAGTGTCCCTGGGAACAGCATG---
---CTCAACATCGTGACAGTCCGGTGCGGGCACTGCACAAACCTACTTTCGGTGAGCGCACGTCCTCCTGAG---
AAGAGGCAGCGTGTTCCTTCGGCGTACAACAGATTCATCAAGGAAGAGATACGAAGGATCAAAGCAAACAACCCC
GACATTAGCCACAGGGAAGCTTTCAGCACTGCCGCAAAGAACTGGGCACATTATCCAAACATCCATTTCGGTCTA
Triticum_aestivum_EU099585
GAGCATGTCTGCTACGTGCGCTGCAACTTCTGCAACACAATTCTCGCGGTCAGTGTTCCAAGTAATAGCATG---
---CTGAACATCGTCACCGTCCGTTGCGGGCACTGCACTAGCCTGCTGTCGGTGAACATGAGACCTCCTGAG---
AAGAGGCAGCGGGTACCTTCAGCGTATAACCGATTTATTAAGGAGGAGATACGAAGGATAAAAACAAACAATCCT
GACATAAGCCACAGAGAAGCCTTCAGCACCGCAGCAAAGAATTGGGCGCATTTTCCAAACATTCATTTCGGGCTA
Triticum_aestivum_AB470269
GAGCACCTCTGCTACGTGCGCTGCACGTACTGCAACACCGTGCTCGCGGTTGGGGTTCCATGCAAGAGGCTG---
---ATGGACACGGTGACTGTGAAATGCGGCCACTGCAACAAC---CTCTCCTTTCTCGTGAAGCCCCCAGAG---
AAGAAACATCGCCTACCATCTGCCTACAATCGCTTCATGAGGGAGGAAATACAACGTATCAAAGCTGCAAAGCCA
GACATCCCTCACAGAGAAGCCTTCAGCATGGCTGCTAAGAACTGGGCGAAGTGCGACCCTCGCTGCTCATCGACT
Triticum_aestivum_AY330228
GAGCAGCTCTGCTACGTGCACTGCCACTTCTGCGACACCGTCCTCGTCGTGAGCGTGCCGAGCAGCAGCCTC---
---TTCAAGACGGTGACAGTGCGGTGCGGCCACTGCAGCAGCCTGCTCACCGTCGACAACAGACCTCCGGAG---
AAGAGGCAGCGCGTGCCGTCGGCGTACAACCGCTTCATCAAGGACGAAATCCAACGCATCAAGGCTGGCAATCCC
GACATCTCGCACAGGGAGGCCTTCAGCGCGGCTGCCAAGAACTGGGCTCACTTTCCGCATATCCACTTTGGCCTC
Welwitschia_mirabilis_DT583102
GAGCAATTGTGCTACGTTCCTTGCACAAACTGCAGCACCGTTCTAGCGGTCAGTGTACCGCTGAAGAGCTTG---
---TTTAAGGTCGTTACAATCAGGTGTGGACACTGCAACAATCTCATATCAGTTGACCCAAGAGCTCCCGAG---

AAAAAACAACGCATTTCTTCTGCATATAATCGATTTATCAAGGAAGAGATTCAGAGGATTAAAGCAGGCAACCCT
AATATGAGCCACCGGGAAGCTTTCAGCACAGCTGCTAAGAACTGGGCTCTTTTCCCCTACATGCAATTAGGCCTC
Zantedeschia_aethiopica_AJ700396
GAGCATGTCTGCTACGTCCACTGCAACTTCTGCAACACCATCTTGGCGGTTAGCGTCCCATCCAACAACTTA---
---TTCAACATTATCACGGTGAGATGCGGGCACTGTTCTAATCTGTTGTCTGTCAATATGCGCCCTCCTGAA---
AAGAGGCAACGTGTTCCATCTGCGTATAACAGGTTTATTAAGGAGGAGATACAAAGGATAAAGGCCAATAACCCG
GATATTAGCCATAGGGAAGCTTTCAGTGCTGCAGCAAAGAATTGGGCTCATCTTCCTCACATCCACTTCGGGCTT
Zea_mays_AY103851
GACCATGTGTGCTATGTGCACTGCAACTTCTGCAACACAGTTCTCGCGGTGAGTGTCCCGGGGAACAGCATG---
---CTCAGCATGGTGACAGTTCGGTGTGGGCACTGCACAAATCTACTGTCAGTGAATCGTCCGGCTCCTGAG---
AAGAGGCAGCGGGTTCCGTCGGCGTATAACAGATTCATCAAGGAAGAGATTCGCAGGATCAAGGCGAACAACCCC
GACATTAGCCACAGGGAAGCCTTCAGCACAGCAGCCAAGAACTGGGCACATTATCCGAACATCCATTTCGGCTTA
Zea_mays_NM001155258
GAGCACCTGTGCTACGTCCGCTGCACCTACTGCAACACCGTGCTCGCGGTTGGGGTTCCATGCAAGAGGCTG---
---ATGGACACGGTGACTGTCAAGTGCGGCCACTGCAACAAC---CTCTCCTACCTCGTCAAGCCCCCGGAG---
AAGAAACACCGCCTCCCATCTGCTTATAATCGCTTCATGAGGGAGGAGATTCAGCGCATCAAAGCTGCGAAGCCA
GATATCCCTCACAGGGAGGCCTTCAGCATGGCTGCCAAGAATTGGGCAAAGTGTGACCCGCGCTGCTCGACGGCT
Zea_mays_AY313904
GAGCAGCTCTGCTACGTGCACTGCCACTTCTGCGACACCGTCCTCGTCGTGAGCGTGCCTACGAGCAGCCTG---
---TTCAAGACGGTGACGGTACGATGCGGCCACTGCAGCAGCTTGCTCACCGTCAACAACAGACCTCCGGAG---
AAGAGGCAGCGCGTGCCGTCGGCGTACAACCGCTTCATCAAGGACGAAATCCAACGCATCAAGGCTGGCAATCCC
GACATCTCGCACAGGGAGGCCTTCAGCGCGGCGGCCAAGAACTGGGCGCACTTTCCACACATCCACTTTGGACTC
Zea_mays_AY313901
GAGCAGATCTGCTACGTGCACTGCAGCTACTGCGACACCATCCTCGCGGTGGGCGTGCCGTGCAGCAGCCTG---
---TTCCAGACGGTCACCGTGCGGTGCGGCCACTGCGCCAACCTGCTCTACGTCAACAACAAGACTTCAGAG---
AAGCGGCAGAGAGTTCCTTCCGCATACAACCGTTTCATCAAAGATGAGATCCAGCGCATCAAGGCCAGCAATCCC
GACATCACTCACAGGGAAGCTTTCAGCGCGGCTGCCAAGAATTGGGCCCATTTCCCACACATCCATTTCGGCCTC
Zea_mays_AY313902
GAGCAGCTCTGCTACGTGCACTGCAACTGCTGCGACACCATCCTCGCCGTCGGCGTGCCTTGCAGCAGCCTG---
---TTCAAGACGGTGACGGTGCGTTGTGGCCACTGCGCCAACCTACTCTCCGTCAACAACAGGCCTCCGGAG---
AAGCGGCAGAGAGTCCCGTCGGCATACAACCGGTTCATCAAGGACGAGATCCAGCGCATCAAGGCCGGCAACCCG
GACATCACCCACCGGGAGGCGTTCAGCGCAGCCGCAAAGAATTGGGCCCATTTCCCACACATCCACTTCGGCCTC
Zea_mays_AY313903
GAGCAGCTCTGCTACGTGCACTGCTACTTCTGCGACACCGTCCTCGTCGTGAGCGTGCCTACGAGCAGCTTG---
---TTCAAGACGGTGACGGTACGATGCGGCCACTGCAGCAGCTTGCTCACCGTCGACAACAGACCTCCGGAG---
AAGAGGCAGCGCGTGCCGTCGGCCTACAACCGCTTCATCAAGGACGAAATCCAACGCATCAAGGCTGGCAATCCC
AACATCTCGCACAGGGAGGCCTTCAGCGCGGCGGCCAAGAACTGGGCGCACTTTCCACATATCCACTTTGGACTC
