# Supplementary figures and images for: Loss of YABBY2-Like Gene Expression May Underlie the Evolution of the Laminar Style in Canna and Contribute to Floral Morphological Diversity in the Zingiberales
Source: Front Plant Sci. 2015 Dec 16;6:1106. doi: 10.3389/fpls.2015.01106 (PMC4679924; doi:10.3389/fpls.2015.01106)

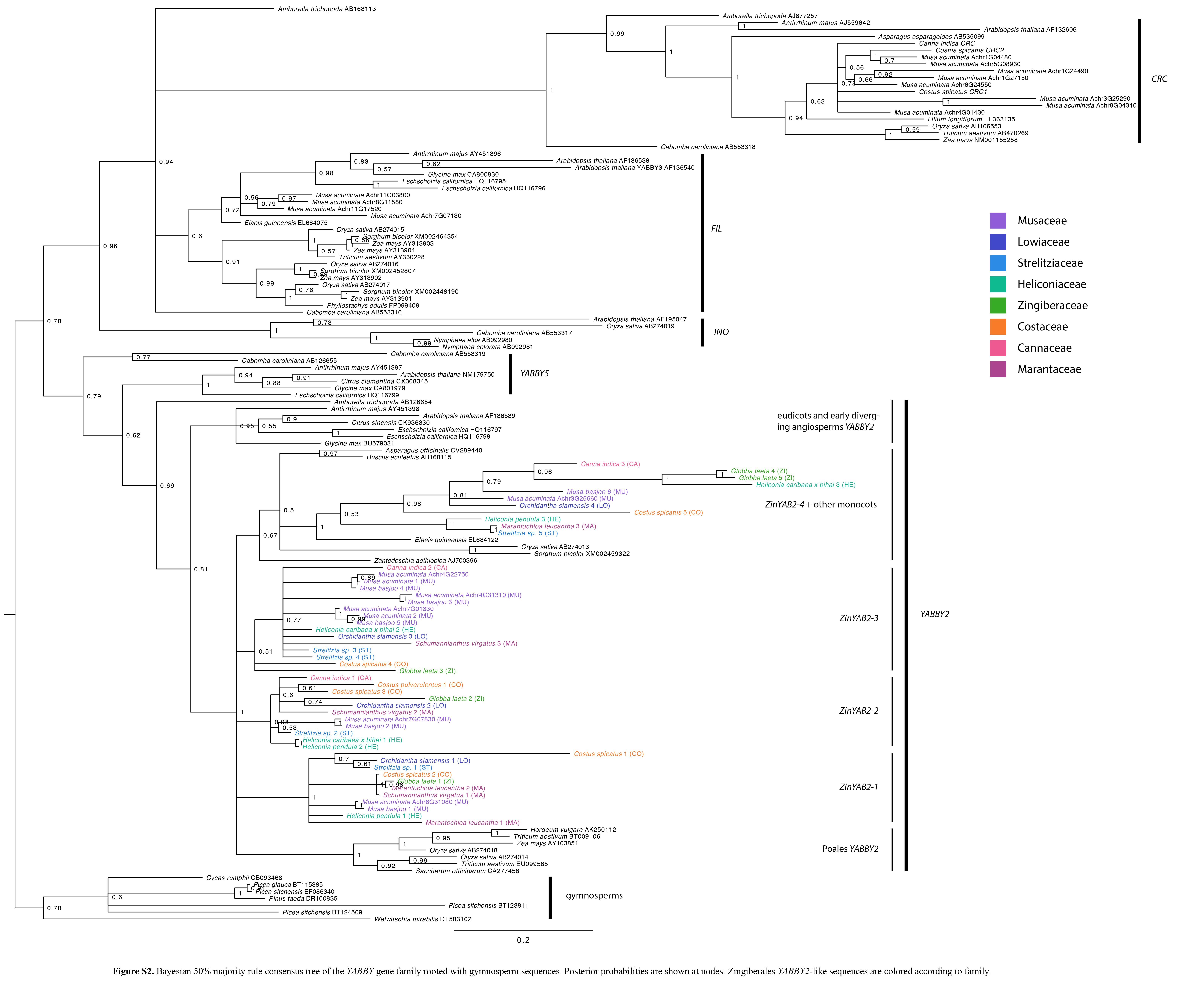

Supplement: Supplementary file 3 [file Image2.jpg]
